# The natural history of intravascular lymphomatosis

**DOI:** 10.1002/cam4.269

**Published:** 2014-06-14

**Authors:** Ekokobe Fonkem, Edwin Lok, David Robison, Shiva Gautam, Eric T Wong

**Affiliations:** 1 Brain Tumor Center and Neuro-Oncology Unit, Department of Neurology, Beth Israel Deaconess Medical CenterBoston, Massachusetts; 2 Division of Biostatistics, Department of Medicine, Beth Israel Deaconess Medical CenterBoston, Massachusetts

**Keywords:** Intravascular lymphomatosis, natural history, non-Hodgkin's lymphoma

## Abstract

Intravascular lymphomatosis (IVL) is a rare and clinically devastating form of extranodal B-cell non-Hodgkin's lymphoma. We performed a comprehensive analysis of the literature on IVL's published between 1959 and 2011 and evaluated the natural history as well as identified prognostic and predictive factors in patients. Nonparametric two-tailed Mann–Whitney *U-*test and Mantel–Cox log rank test were used to evaluate the survival intervals and prognostic factors. Multivariate analysis of variance (MANOVA) and chi-squared statistics were carried out to examine treatment-related predictive factors. Of the 740 patients with IVL, 651 (88%) had a diagnosis of B-cell lymphoma, 45 (6%) with T-cell lymphoma, and 12 patients (2%) with NK cell lymphoma. Central nervous system (CNS) IVL had the highest proportion of postmortem diagnosis, 250 (60%) compared to 21 (8%) of skin, 28 (11%) of bone marrow (BM) and spleen, and 17 (7%) of lung IVL's. Age <70 years (*P* = 0.0073), non-CNS site of initial diagnosis (*P* = 0.0014), lactate dehydrogenase (LDH) <700 (*P* = 0.0112), and rituximab treatment (*P* < 0.0001) were favorable prognostic factors. Gender, ethnicity, hemoglobin, BM biopsy, and the type of imaging studies used were not significant. Rituximab and doxorubicin treatment worked significantly better in patients with age >71 and LDH >577 compared to nonrituximab, nondoxorubicin regimens (MANOVA 2 degrees of freedom, *P* = 0.0345), with a median time from treatment to death of 20.0 (95% confidence interval [CI] 14.0–N/A, *n* = 14) months versus 2.0 (95%CI 0.5–N/A, *n* = 5) (*χ*^2^ = 4.7, *P* = 0.0304). Patients with CNS IVL relapsed primarily in the CNS (88%) while same-organ relapse occurred less frequently in skin (23%), BM and spleen (50%) and lung (20%) IVL's. Our results indicate that IVL is primarily a disease of B-lymphoma cells. Timely diagnosis and treatment with rituximab-based chemotherapy improve patient survival. The pattern of recurrence is different between CNS IVL and IVL's in other organs.

## Introduction

Intravascular lymphoma (IVL) is a rare and infrequent form of systemic extranodal non-Hodgkin's lymphoma characterized by the proliferation and aggregation of large B-lymphoma cells within the lumen of small blood vessels 1. The peculiar affinity of lymphoma cells to the intravascular space is due to the absence of CD29 (*β*1 integrin) and CD54 (ICAM-1) surface ligands, which may disable them from diapedesis across the endothelium 2. IVL can affect virtually any organ in the body, but most commonly involves the central nervous system (CNS) and skin, but organs like lungs, bone marrow (BM), and spleen are also targets 1. This disorder generally has a rapidly fatal outcome, with patient survival lasting only a few months 1. This is primarily due to a lack of BM involvement or enlarged lymph nodes, making biopsy of affected tissue for diagnosis a particularly challenging endeavor. Furthermore, patients with CNS IVL present with stroke-like symptoms and this disorder is often not included in the differential diagnosis. As a result, the diagnosis of IVL is made postmortem in over 50% of the cases 3,4. Furthermore, unlike primary CNS lymphoma with which a high responses rate is usually seen, the treatment of IVL remains suboptimal due to the rarity of this disorder and the difficulty to establish a diagnosis in a timely fashion. Although timely administration of systemic chemotherapy can induce remission, there is a lack of prospective study that documents the natural history, prognostic factors, and treatment efficacy to guide physician practice. Because published literature consists primarily of case reports and retrospective case series, we performed a meta-analysis of existing literature to address these questions.

## Methods

### Literature search

We conducted a comprehensive literature search of the electronic databases Medline/PubMed, Paperchase and Web of Science (from 1962 to December, 2011) using the following search terms: intravascular with the term lymphomatosis or lymphoma; angioendotheliomatosis with and without the term malignant; endotheliomatosis with and without the term systemic; and large cell lymphoma with the terms angiotrophic or angiotropic. Multiple primary subject heading search terms were used because of a change in nomenclature from malignant angioendotheliomatosis to intravascular lymphomatosis, which occurred in 1986 5,6. Furthermore, we reviewed reference lists of retrieved publications to search for more case reports. Only case reports that were written in English and reported on human patients were considered.

### Inclusion and exclusion criteria

For inclusion, case reports had to fulfill the following criteria: contained sufficient clinical and survival data; reported on human patients diagnosed ante- or postmortem with IVL; and written in English. Case reports were excluded if: diagnosis, disease progression, and survival data were insufficient for analysis; the publication was written in non-English language; or they were veterinary publications. If multiple published reports describe the same patient, we included only the one with the most detailed information.

### Data extraction

Data were extracted independently by one investigator (DR) and were independently verified by the primary investigators (EF and ETW) for accuracy. For each publication, the following information was extracted: first three author's last names; year of publication; age; gender; Asian or non-Asian ethnicity; immuno-histological markers used to confirm diagnosis; affected organs; laboratory values: white blood cell ( WBC) count, hemoglobin, and lactate dehydrogenase (LDH); treatments; and survival times, which were divided into four epochs: (1) time from initial presentation of symptoms to death, (2) time from diagnosis to death, (3) time from treatment to death, and (4) time from treatment to first recurrence.

### Statistical methods

IVL natural history and prognostic factors were evaluated using nonparametric two-tailed Mann–Whitney *U*-test and Mantel–Cox Log Rank test to investigate differences in patient survival outcome according to age, gender, and ethnicity. The same method was also applied to groups according to organ site of first IVL presentation (i.e., CNS, skin, lungs, etc.) and the median, range, and *P* value were calculated and presented graphically. Laboratory prognostic factors were grouped as follows: WBC as normal (4–12 × 10^9^/L) versus abnormal (<4 or ≥12 × 10^9^/L), hemoglobin < or ≥10.5 g/dL, and LDH < or ≥700 IU/L. Patient outcome and treatment response were also analyzed according to the respective imaging techniques used for diagnosis and the chemotherapy regimen received.

To control for prognostic variables found to be significant in the initial analysis (age and LDH), which were possibly contributing to better patient treatment outcome when treated with rituximab and doxorubicin, a Kaplan–Meier regression was applied to the two groups according to treatment type (rituximab and doxorubicin versus nonrituximab and nondoxorubicin). Data were first read into the R statistical package (www.r-project.org) database as an array (age, LDH, time to death, censor status, treatment type), and a summary of statistics was generated that included the median and the 95% confidence interval (CI). Multivariate analysis of variance (MANOVA) was used to analyze the influence of predictive factors on treatment. A chi-squared test for independence between the two groups was computed and a Kaplan–Meier survival plot was then generated.

A three-dimensional analysis was performed to investigate possible interaction of age and LDH as predictive factors on patients treated with rituximab and doxorubicin. First, data on age, LDH, and time of death were read into R database as an array. Patients with unreported data variables were filtered out and test cut-off points were recursively determined by setting bounds at all possible combinations of the supplied data, using age and LDH as the test coordinates. Second, *P* values were computed based on the Wilcoxon rank sum test for time to death values in patients as defined by the age and LDH boundaries. Lastly, a three-dimensional plot of all the data points included in the “optimal” cut-off determination was generated and the appropriate cutoffs illustrated as orthogonal planes.

## Results

**Figure 1:**
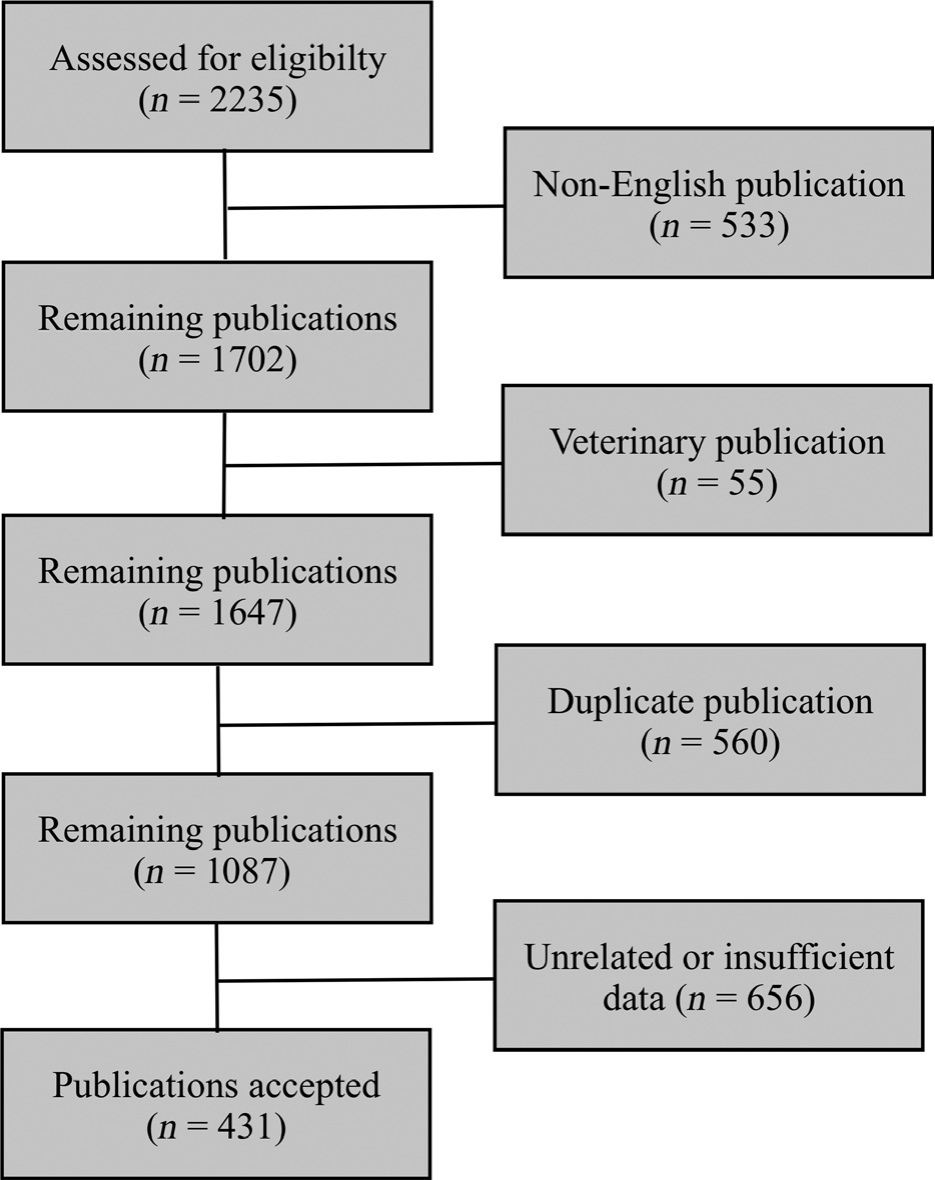
Flow diagram of inclusion criteria. A systematic search was performed using Medline/PubMed, Paperchase and Web of Science by applying keywords of intravascular lymphoma, intravascular lymphomatosis, angioendotheliomatosis, angiotropic/angiotrophic large cell lymphoma, endotheliomatosis, and systemic endotheliomatosis. After accounting for overlap publications between keyword strings, articles were further examined and excluded when they were (1) not written in English, (2) not concerning human subjects, or (3) unrelated to the clinical evaluation of IVL. Data were extracted from the remaining publications and only those containing sufficient information were included in the statistical analysis. IVL, intravascular lymphomatosis.

Using the predefined search criteria, we identified 431 publications (Fig.1) that included 740 IVL patients with sufficient data for extraction. The distribution of publications by decade is as follows: 3 in 1960s, 7 in 1970s, 40 in 1980s, 111 in 1990s, and 270 in 2000s (until December 2011). To account for possible publication bias, the statistical methods outlined above were used to investigate possible significant differences in patient survival across decades.

### Patient characteristics

**Figure 2:**
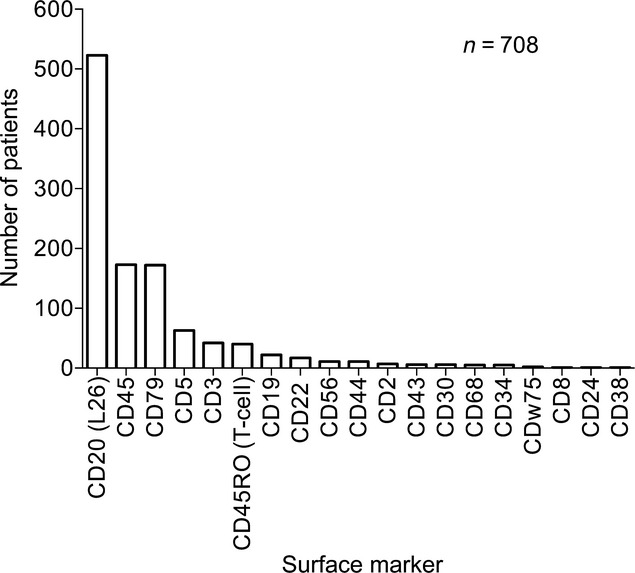
Distribution of surface markers in IVL (*n* = 740). Positive immunohistochemical staining include 523 (71%) CD20, 173 (23%) CD45, 172 (23%) CD79, 63 (9%) CD5, 42 (6%) CD3, 22 (3%) CD19, 17 (2%) CD22, 11 (1%) CD56, and 11 (1%) CD44. Only 40 (5%) had the T-cell marker CD45RO. IVL, intravascular lymphomatosis.

Cases of IVL (*n* = 740) were compiled from 431 publications published between 1959 and 2011 (Tables S1A and S1B) and patient characteristics are listed in Table1. The median age was 64 (range 0.4–90.0) years and gender distribution was equal. There were 312 (42%) Asians and 428 (58%) non-Asians. Pathology data were available for 708 patients and the majority of the IVL's were of B-cell origin 651 (88%) and only 45 (6%) and 12 (2%) were of T-cell and NK-cell origin, respectively. The distribution of surface markers by immunohistochemical staining revealed that 523 cases (71%) were positive for CD20, 173 (23%) positive for CD45, 172 (23%) positive for CD79, 40 (5%) positive for CD45RO, and 63 (9%) positive for CD5 (Fig.2).

**Table 1:** Baseline patient demographic and clinical characteristics.

Characteristics	Number
All patients	740
*Age, years*	
Median	64
Range	0.4–90
Gender	
Males	379
Females	354
Unknown	7
Ethnicity	
Asian	312
Non-Asian	428
Pathology	
B Cell	651
T Cell	45
NK cell	12
Unknown	32
Primary site of disease	
CNS	308
Skin	145
BM and/or spleen	124
Lungs	50
Kidney	18
Adrenal	17
Spine	11
Liver	10
Abdomen	9
Prostate	8
Uterus	6
Eye	3
Muscle	3
Intestine	2
Skeletal muscle	2
Other	24
Survival (months)	Median (range)
Initial symptoms to death	5.0 (0.7–96.0)
Diagnosis to death	7.0 (0.1–165.0)
Treatment to death	9.0 (0.1–165.0)
Treatment to first recurrence	6.0 (0.3–125.0)

**Figure 3:**
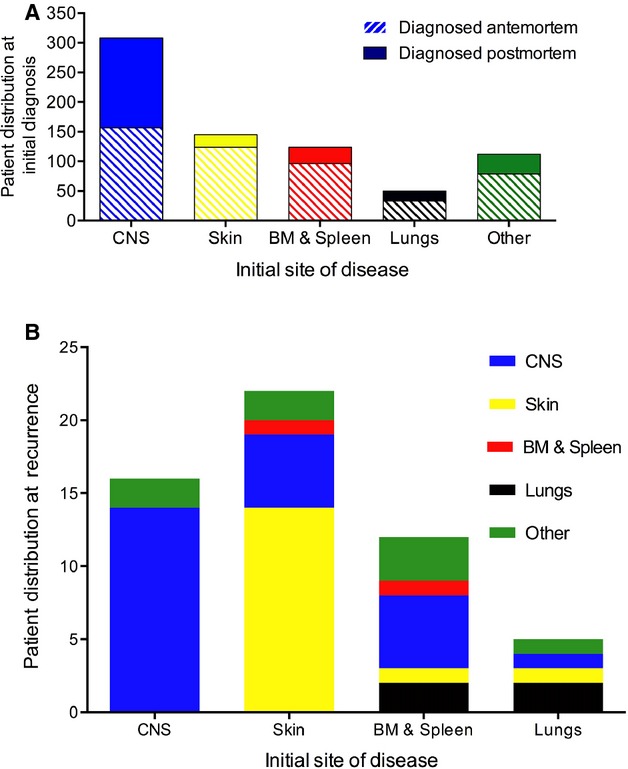
Patient distribution at initial diagnosis and at recurrence. (A) Among the newly diagnosed IVL patients, 308 (41%) presented in the CNS, 145 (20%) in skin, 124 (17%) in BM and spleen, 50 (7%) in lungs, 101 (13%) in other sites and 12 (2%) unknown. Among the 250 patients diagnosed at postmortem, IVL affecting the CNS was highest compared to other organ sites: 151 (60%) CNS, 21 (8%) skin, 28 (11%) BM and spleen, and 17 (7%) lung and 33 (13%) other organs. (B) Same-organ recurrence was highest in 14 of 16 (88%) CNS, as compared to 5 of 22 (23%) skin, 5 of 10 (50%) BM and spleen, and 1 of 5 (20%) lung IVL's. BM, bone marrow; CNS, central nervous system; IVL, intravascular lymphomatosis.

The initial organ affected is presented in Figure3A. IVL was first diagnosed in 308 (41%) patients in the CNS, 145 (20%) in skin, 124 (17%) in BM and spleen, and 50 (7%) in the lungs. Diagnosis was established in 250 patients (34%) at postmortem. For CNS IVL, timely diagnosis is challenging because 151 (60%) were made only at postmortem compared to 21 (8%) of skin, 28 (11%) of BM and spleen, and 17 (7%) of lung IVL's.

### Prognostic factors

**Figure 4:**
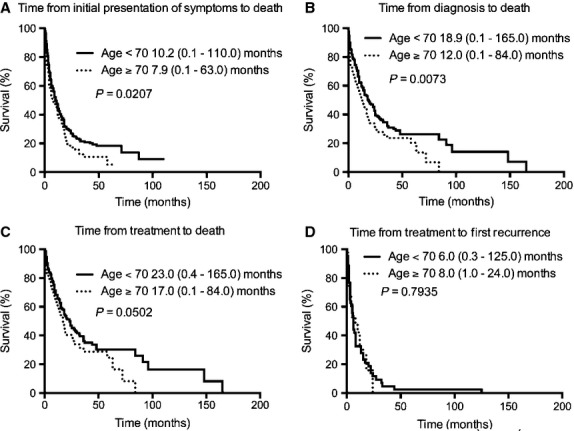
Kaplan–Meier survival curves according to age. Four epochs were examined according to time from (A) initial presentation of symptoms to death, (B) time from diagnosis to death, (C) time from treatment to death, and (D) time from treatment to first recurrence.

We analyzed factors that would prognosticate patient outcome by comparing the survival time from diagnosis to death with respect to clinical and laboratory parameters. We chose this time interval because both diagnosis and death dates are discrete time points and most frequently reported. Patients had significantly shorter survival when they had age ≥70 years (*P* = 0.0073), CNS disease (*P* = 0.0014), and serum LDH ≥700 IU/L (*P* = 0.0112) (Table2). Furthermore, the same age effect is also seen with respect to time from initial presentation of symptoms to death (*P* = 0.0207), time from diagnosis to death (*P* = 0.0073), and time from treatment to death (*P* = 0.0502); but not time from treatment to first recurrence (*P* = 0.7935) (Fig.4). The prognostic power of gender, ethnicity, non-CNS presentation, WBC, hemoglobin, and imaging modality used did not reach significance (Table2).

**Table 2:** Prognostic characteristics for intravascular lymphomatosis.

Clinical prognostic factor	Time from diagnosis to death	*P*
Age, years		
<70	18.9 (0.1–165.0)	0.0073*
≥70	12.0 (0.1–84.0)	
Gender		
Male	9.3 (0.1–165.0)	0.6615
Female	10.0 (0.1–96.0)	
Ethnicity		
Asian	10.0 (0.1–165.0)	0.0760
Non-Asian	9.0 (0.1–96.0)	
Primary site of disease		
Non-CNS	19.0 (0.1–165.0)	
CNS	14.0 (0.1–84.0)	0.0014*
1960–1980′s	6.0 (0.9–14.0)	
1990's	8.0 (0.2–71.0)	0.4187*
2000's	18.0 (0.3–84.0)	0.0462*
Non-Skin	9.3 (0.1–165.0)	
Skin	11.0 (0.1–96.0)	0.3802
1960–1980's	19.9 (2.1–42.0)	
1990's	17.0 (0.3–63.0)	0.9185*
2000's	24.0 (0.1–96.0)	0.6318*
Non-BM & Spleen	9.0 (0.1–165.0)	
BM & Spleen	11.5 (0.1–148.0)	0.0830
1990's	18.0 (0.1–36.0)	
2000's	17.0 (0.1–148.0)	0.7102
Non-Lungs	10.0 (0.1–165.0)	
Lungs	9.0 (0.1–96.0)	0.7714
1990's	9.0 (0.5–96.0)	
2000's	29.0 (0.1–48.0)	0.7904*
WBC (×10^9^/L)		
Normal	10.0 (0.1–92.0)	0.6101
Abnormal	9.5 (0.1–165.0)	
Hgb		
<10.5 g/dL	10.0 (0.1–148.0)	0.9471
≥10.5 g/dL	10.0 (0.1–165.0)	
LDH		
<700 IU/L	15.0 (1.0–165.0)	0.0112
≥700 IU/L	9.0 (0.1–148.0)	
Imaging		
MRI	10.0 (0.1–165.0)	0.2551
CT	9.2 (0.1–165.0)	0.5043

* *P* values represent results of Mantel Cox log rank test; all remaining *P* values represent results of Mann–Whitney *U* test.

### Outcomes of CNS IVL

**Figure 5:**
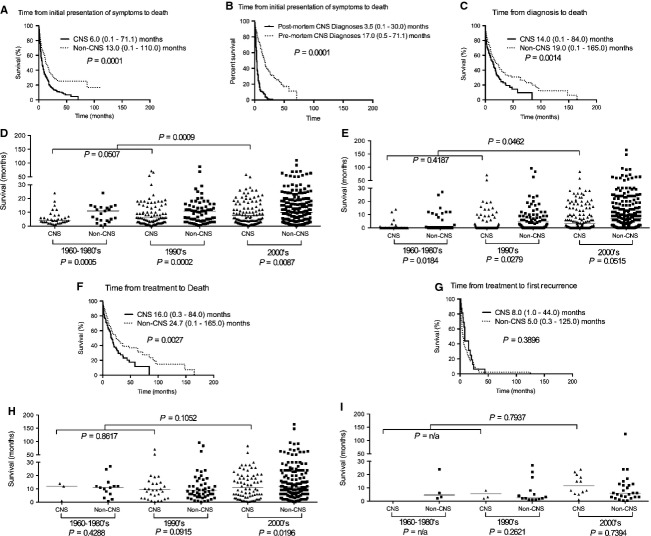
Kaplan–Meier survival curves for CNS versus non-CNS IVL patients. Patients with CNS IVL had significantly shorter survival than non-CNS patients in (A) time from initial presentation of symptoms to death, (C) time from diagnosis to death, and (F) time from treatment to death, but not (G) time from treatment to first recurrence. (B) The median survival of CNS IVL patients diagnosed postmortem was significantly shorter than those diagnosed premortem, 3.5 (range 0.1–30.0, *n* = 150) versus 17.0 (range 0.5–71.1, *n* = 98) months, respectively (*P* = 0.0001). (D) The difference between CNS and non-CNS IVL remains significant in each successive decade for median time from initial presentation of symptoms to death in the 1960–1980s (CNS versus non-CNS: 4.1 [range 1.0–24.0, *n* = 35] versus 12.0 [range 0.3–33.0, *n* = 23] months [*P* = 0.0005]), 1990s (CNS versus non-CNS: 6.0 [range 0.1–71.0, *n* = 91] versus 12.0 [range 0.4–87.0, *n* = 75] months [*P* = 0.0002]), and 2000s (CNS versus non-CNS: 9.0 [range 0.1–58.0, *n* = 122] versus 14.0 [range 0.1–110.0, *n* = 206] months [*P* = 0.0087]). For CNS IVL, the difference was also significant between decades 1960–1980s and 1990s (*P* = 0.0507) and 1960–1990s and 2000s (*P* = 0.0009). (E) For median time from diagnosis to death, the difference between CNS and non-CNS IVL remains significant in each successive decade in the 1960–1980s (CNS versus non-CNS: 6.0 [range 0.9–14.0, *n* = 6] versus 15.0 [range 0.5–42.0, *n* = 14] months [*P* = 0.0184]) and 1990s (CNS versus non-CNS: 8.0 [range 0.1–71.0, *n* = 43] versus 17.0 [range 0.1–96.0, *n* = 58] months [*P* = 0.0279]), and near significance in 2000s 18.0 [range 0.3–84.0, *n* = 98] versus 20.0 [range 0.1–165.0, *n* = 221] months [*P* = 0.0515]. For CNS IVL, the difference was only significant between decades 1960–1990s versus 2000s (*P* = 0.0462). (H) For time from treatment to death, only the decade 2000s was significant for CNS versus non-CNS IVL: 20.0 (range 0.3–84.0, *n* = 82) versus 30.0 (range 0.1–165.0, *n* = 171) months (*P* = 0.0196) but not significant for CNS IVL's in successive decades. (I) For time from treatment to first recurrence, no statistical difference was found in survival in CNS versus non-CNS, as well as CNS IVL's, in each successive decades. IVL, intravascular lymphomatosis; CNS, central nervous system.

We analyzed the four time epochs outlined in the Methods section to confirm the prognostic factors and to obtain a more detailed view of patient survival when IVL was presented in various organs (Fig.5). Patients with CNS IVL had significantly shortened survival in 3 of 4 time epochs. Compared to non-CNS IVL, CNS IVL had a shorter median time from initial presentation of symptoms to death (13.0 [range 0.1–110.0, *n* = 304] versus 6.0 [range 0.1–71.1, *n* = 248] months, respectively [*P* = 0.0001]), median time from diagnosis to death (19.0 [range 0.1–165.0, *n* = 293] versus 14.0 [range 0.1–84.0, *n* = 141] months, respectively [*P* = 0.0014]), and median time from treatment to death (24.7 [range 0.1–165.0, *n* = 240] versus 16.0 [0.3–84.0, *n* = 117] months, respectively [*P* = 0.0027]). Because a high proportion of CNS IVL patients were diagnosed postmortem, we therefore analyzed the survival of patients diagnosed postmortem versus premortem and found a significant difference, 3.5 (range 0.1–30.0, *n* = 150) versus 17.0 (range 0.5–71.1, *n* = 98) months, respectively (*P* = 0.0001). There was no difference between non-CNS and CNS IVL patients with respect to median time from treatment to first recurrence.

Because improvements in the diagnosis and treatment of non-Hodgkin's lymphoma have been made over the past decades, we asked whether or not the survival of patients with CNS IVL would also improve in successive decades. We grouped patients according to 1960–1980s, 1990s, and 2000s because of the pivotal advancements in the pathological diagnosis of this disease as intravascular lymphomatosis in the 1980s, utilization of doxorubicin-based cytotoxic chemotherapy in 1990s, and addition of rituximab in 2000s. Compared to the 1960–1990s, there was improvement in the time from diagnosis to death in the 2000s (6.0 [range 0.9–14.0, *n* = 6] versus 18.0 [range 0.3–84.0, *n* = 98] months, respectively [*P* = 0.0462]) but not between 1960–1980s and 1990s (Table2).

**Figure 6:**
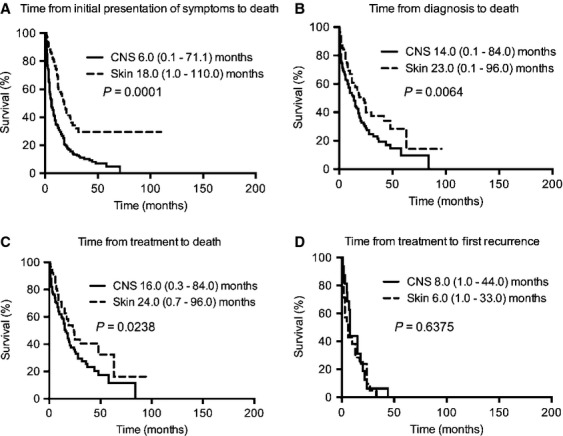
Kaplan–Meier survival curves for CNS versus skin IVL patients. Compared to skin IVL, CNS IVL had a significantly shorter (A) median time from initial presentation of symptoms to death (18.0 [range 1.0–110.0, *n* = 112] versus 6.0 [range 0.1–71.1, *n* = 248] months, respectively [*P* = 0.0001]), (B) median time from diagnosis to death (23.0 [range 0.1–96.0, *n* = 110] versus 14.0 [range 0.1–84.0, *n* = 141] months, respectively [*P* = 0.0064]), and (C) median time from treatment to death (24.0 [range 0.7–96.0, *n* = 94] versus 16.0 [0.3–84.0, *n* = 117] months, respectively [*P* = 0.0238]). There was no difference between skin and CNS IVL patients with respect to (D) median time from treatment to first. recurrence (6.0 [range 1.0–33.0, n = 21] versus 8.0 [1.0–44.0, n = 16] months, respectively [*P* = 0.6375]). IVL, intravascular lymphomatosis; CNS, central nervous system.

**Figure 7:**
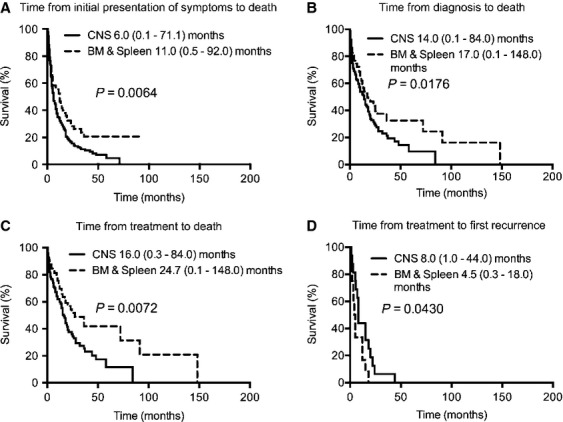
Kaplan–Meier survival curves for patients with IVL in the CNS versus BM and spleen. Compared to IVL in the BM and spleen, CNS IVL also had a significantly shorter (A) median time from initial presentation of symptoms to death (11.0 [range 0.5–92.0, *n* = 80] versus 6.0 [range 0.1–71.1, *n* = 248] months, respectively [*P* = 0.0064]), (B) median time from diagnosis to death (17.0 [range 0.1–148.0, *n* = 92] versus 14.0 [range 0.1–84.0, *n* = 141] months, respectively [*P* = 0.0176]), (C) median time from treatment to death (24.7 [range 0.1–148.0, *n* = 65] versus 16.0 [0.3–84.0, *n* = 117] months, respectively [*P* = 0.0072]), and (D) median time from treatment to first recurrence (4.5 [range 0.3–18.0, *n* = 12] versus 8.0 [1.0–44.0, *n* = 16] months, respectively [*P* = 0.0430]). IVL, intravascular lymphomatosis; CNS, central nervous system; BM, bone marrow.

We next compare the outcome of CNS IVL with other IVL's in specific organs (Figs.6 and 7). Compared to skin IVL, CNS IVL also had a significantly shorter median time from initial presentation of symptoms to death, median time from diagnosis to death, and median time from treatment to death. There was no difference between skin and CNS IVL patients with respect to median time from treatment to first recurrence. Furthermore, compared to IVL in the BM and spleen, CNS IVL also had a significantly shorter median time intervals in all four epochs.

To further clarify if CNS involvement remains a robust but unfavorable prognostic factor for IVL, we analyzed the survival of patients with CNS versus non-CNS IVL in each of the decades (Fig.5). Indeed, compared to those without CNS disease, patients with CNS IVL had significantly shorter median time from initial presentation of symptoms to death in 1960–1980s (*P* = 0.0005), 1990s (*P* = 0.0002), and 2000s (*P* = 0.0087). Likewise, for CNS IVL, there was also a significant shorter median time from diagnosis to death in 1960–1980s (*P* = 0.0184), 1990s (*P* = 0.0279), and 2000s (*P* = 0.0515). However, for median time from treatment to death, there was a trend toward eventual statistical significance from 1960–1980s (*P* = 0.4288), to 1990s (*P* = 0.0915), and to 2000s (*P* = 0.0196). To put into perspective, these data indicate that CNS IVL has an intrinsically poorer prognosis than non-CNS IVL but treatment advances in the 2000s may have accentuated this difference.

### Outcome of IVL in other organ sites

**Figure 8:**
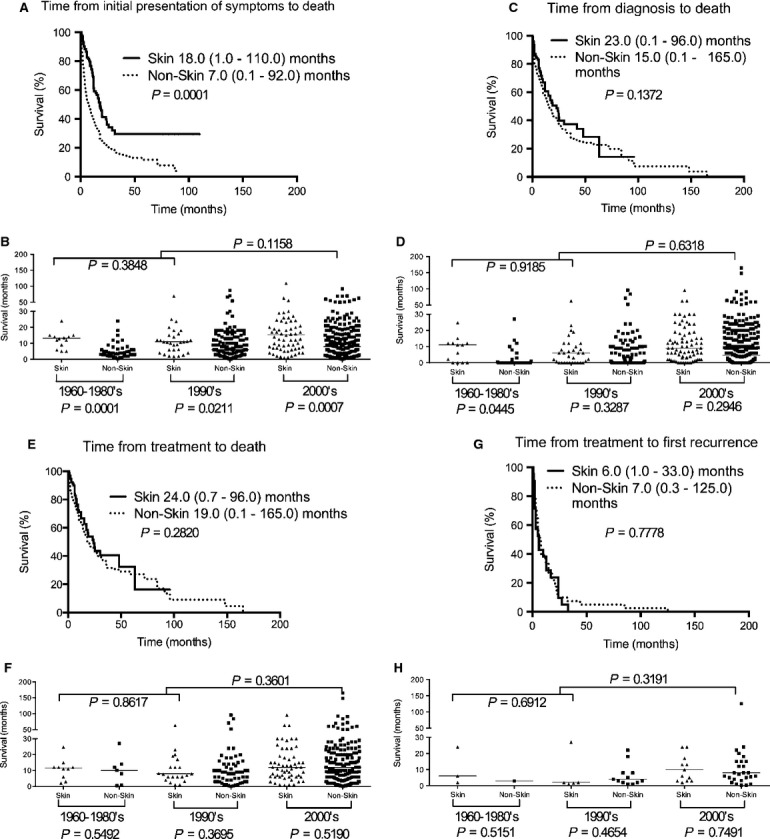
Kaplan–Meier survival curves for skin versus nonskin IVL patients. Patients with skin IVL had significantly better survival than nonskin patients in (A) time from initial presentation of symptoms to death but not (C) time from diagnosis to death, (E) time from treatment to death, or (G) time from treatment to first recurrence. (B) The difference between skin and nonskin IVL remains significant in each successive decade for median time from initial presentation of symptoms to death in the 1960–1980s (skin versus nonskin: 15.0 [range 5.0–31.7, *n* = 14] versus 4.0 [range 0.3–33.0, *n* = 44] months [*P* = 0.0001]), 1990s (skin versus nonskin: 12.0 [range 1.7–69.0, *n* = 32] versus 6.2 [range 0.1–87.0, n = 134] months [*P* = 0.0211]), and 2000s (skin versus nonskin: 20.0 [range 1.0-110.0, *n* = 66] versus 10.0 [range 0.1–92.0, *n* = 262] months [*P* = 0.0007]). For skin IVL, the difference was not significant between decades 1960–1980s and 1990s (*P* = 0.3848) or 1960–1990s and 2000s (*P* = 0.1158). (D) For median time from diagnosis to death in the 1960–1980s, there was significant difference between skin and nonskin IVL: 19.9 (range 2.1–42.0, *n* = 10) versus 10.0 (range 0.5–27.0, *n* = 10) months (*P* = 0.0445). No difference was found in 1990s (*P* = 0.3287) and 2000s (*P* = 0.2946). Comparing skin IVL between decades, the difference was not significant between decades 1960–1980s and 1990s (*P* = 0.9185) or 1960–1990s and 2000s (*P* = 0.6318). (F) For median time from treatment to death, there was no difference between skin and nonskin IVL in the 1960–1980s (*P* = 0.5492), 1990s (*P* = 0.3695), and 2000s (*P* = 0.5190). Comparing skin IVL between decades, there is also no difference between decades 1960-1980s and 1990s (*P* = 0.8617) or 1960–1990s and 2000s (*P* = 0.3601). (H) For median time from treatment to first recurrence, there is no difference between skin and nonskin IVL in the 1960-1980s (*P* = 0.5151), 1990s (*P* = 0.4654), and 2000s (*P* = 0.7491). Comparing skin IVL between decades, there is also no difference between decades 1960-1980s and 1990s (*P* = 0.6912) or 1960-1990s and 2000s (*P* = 0.3191). IVL, intravascular lymphomatosis.

To further clarify clinical outcome in other organs, we obtained a more detailed view of patient survival when IVL was initially presented in skin, BM and spleen, and lungs. Compared to nonskin IVL, IVL in the skin had a significantly longer median time from initial presentation of symptoms to death (7.0 [range 0.1–92.0, *n* = 440] versus 18.0 [range 1.0–110.0, *n* = 112] months respectively [*P* = 0.0001]) but not time from diagnosis to death (*P* = 0.1372), time from treatment to death (*P* = 0.2820), or time from treatment to first recurrence (*P* = 0.7778) (Fig.8). Furthermore, in each successive decade, the median time from initial presentation of symptoms to death remained significantly prolonged in skin versus nonskin IVL in 1960-1980s (*P* = 0.0001), 1990s (*P* = 0.0211), and 2000s (*P* = 0.0007), while the median time from diagnosis to death was marginally significant in 1960–1980s (*P* = 0.0445) but insignificant in the 1990s (*P* = 0.3287) and 2000s (*P* = 0.2946). The median time from treatment to death in successive decades was not significant either. Taken together, IVL involving the skin has a more favorable prognosis than other organ sites. The data also suggest that our ability to diagnose skin IVL remained unchanged in successive decades while improvements most likely made a difference in nonskin IVL causing a progressive increase in the median time from presentation of symptoms to death and median time from diagnosis to death.

**Figure 9:**
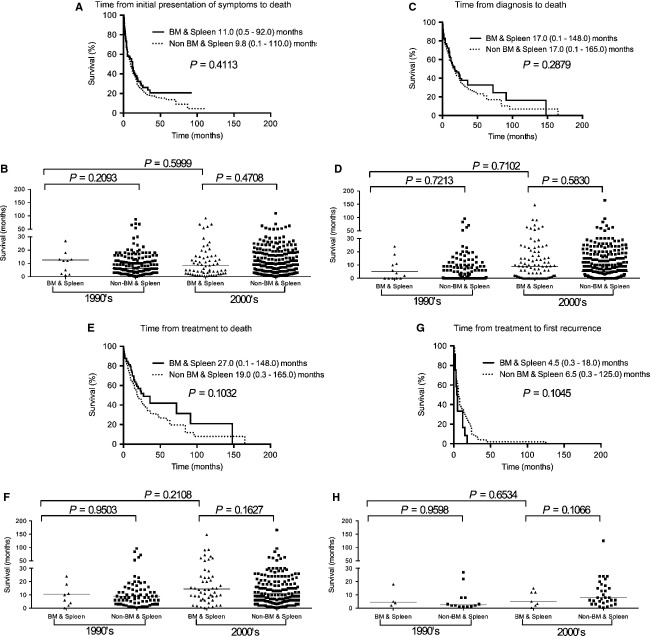
Kaplan–Meier survival curves for BM and spleen versus non-BM and spleen IVL patients. No statistical difference in patient survival between BM and spleen IVL and non-BM and spleen IVL with respective to (A) time from initial presentation of symptoms to death, (C) time from diagnosis to death, (E) time from treatment to death or (G) time from treatment to first recurrence. The difference is also not significant between the two groups in the 1990s and 2000s, as well as BM and spleen IVL between 1990s and 2000s, for (B) median time from initial presentation of symptoms to death, (D) median time from diagnosis to death, (F) median time from treatment to death, and (H) median time from treatment to first recurrence. IVL, intravascular lymphomatosis; BM, bone marrow.

There was also no significant difference between BM and spleen versus non-BM and spleen (Fig.9) as well as lung versus nonlung IVL (Fig.10). In the successive decade analysis of these two organs, there were not enough cases for statistics to be performed in 1960–1980s; but for 1990s and 2000s there was no significance in the time from presentation of symptoms to death, time from diagnosis to death, or time from treatment to death.

**Figure:**
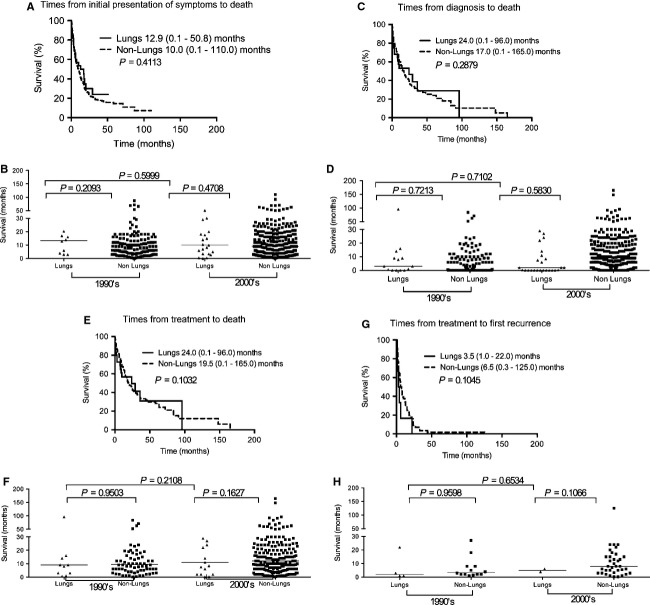
Kaplan–Meier survival curves for lung versus nonlung IVL patients. No statistical difference in patient survival between lung IVL and non-lung IVL with respective to (A) time from initial presentation of symptoms to death, (C) time from diagnosis to death, (E) time from treatment to death or (G) time from treatment to first recurrence. The difference is also not significant between the two groups in the 1990s and 2000s, as well as lung IVL between 1990s and 2000s, for (B) median time from initial presentation of symptoms to death, (D) median time from diagnosis to death, (E) median time from treatment to death, and (H) median time from treatment to first recurrence. IVL, intravascular lymphomatosis.

### Treatment outcome

We next analyzed the effect of chemotherapy on patient outcome by evaluating the time of treatment to death and to first recurrence with respective to antilymphoma drugs such as doxorubicin, methotrexate, and rituximab (Table3). Patients who received rituximab had more than double the median time of treatment to death than those without, 36.0 (range 0.1–72.0, *n* = 115) versus 15.0 (range 0.1–165.0, *n* = 244) months, respectively (*P* < 0.0001). There was also a trend suggesting benefit from doxorubicin-based versus non-doxorubicin-based regimens, or 24.0 (range 0.1–148.0, *n* = 251) versus 18.0 (range 0.5–165.0, *n* = 105) months respectively (*P* = 0.0907). However, no difference was observed when methotrexate was compared to non-methotrexate-based regimens, 16.0 (range 1.0–44.0, *n* = 32) and 20.0 (range 0.1–165.0, *n* = 326) months, respectively (*P* = 0.1484), most likely due to the small sample size of methotrexate-treated patients.

**Table 3:** Chemotherapy treatment outcome.

Regimen	Treatment to death (months)	*P*^1^	Regimen	Treatment to first recurrence (months)	*P*^1^
Doxorubicin (*n* = 251)	24.0 (0.1–148.0)	0.0907	Doxorubicin (*n* = 62)	7.0 (0.3–125.0)	0.4495
Nondoxorubicin (*n* = 105)	18.0 (0.5–165.0)		Nondoxorubicin (*n* = 22)	8.0 (2.0–27.0)	
Methotrexate (*n* = 32)	16.0 (1.0–44.0)	0.1484	Methotrexate (*n* = 7)	8.0 (1.0–44.0)	0.8900
Nonmethotrexate (*n* = 326)	20.0 (0.1–165.0)		Nonmethotrexate (*n* = 74)	6.0 (0.3–125.0)	
Rituximab (*n* = 115)	36.0 (0.1–72.0)	<0.0001	Rituximab (*n* = 21)	12.0 (1.0–33.0)	0.9531
Nonrituximab (*n* = 244)	15.0 (0.1–165.0)		Nonrituximab (*n* = 66)	6.5 (0.3–125.0)	
Rituximab and doxorubicin (*n* = 103)	36.0 (0.1–72.0)	<0.0001	Rituximab and doxorubicin (*n* = 17)	13.0 (1.0–33.0)	0.8148
Nonrituximab and nondoxorubicin (*n* = 75)	10.0 (0.4–45.0)		Nonrituximab and nondoxorubicin (*n* = 19)	10.0 (1.0–27.0)	

1 *P* values represent results of Mantel–Cox log rank test.

**Figure 11:**
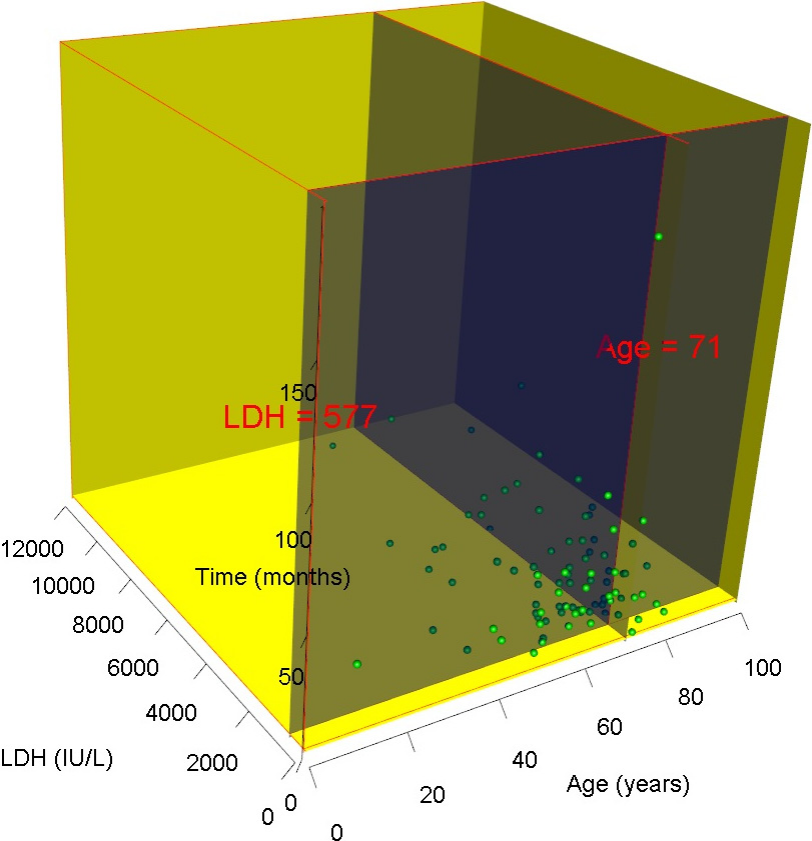
Predictive factors for response to rituximab and doxorubicin. Three-dimensional plot of the MANOVA analysis showing a cut off of age at 71 years and LDH at 577 had the greatest influence on the time from treatment to death (two degrees of freedom, *P* = 0.0345). LDH, lactate dehydrogenase; MANOVA, multivariate analysis of variance.

**Figure 12:**
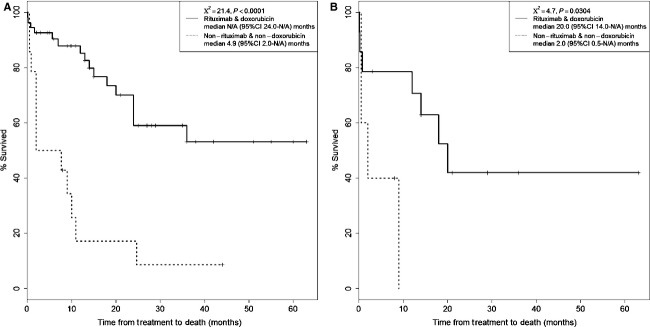
Kaplan–Meier plot of time from treatment to death. (A) Patients with LDH > 577 treated with rituxmab and doxorubicin had a significant prolongation of time from treatment to death, median not reached (95%CI 24.0–N/A, *n* = 14) months, as compared to nonrituximab nondoxorubicin regimens, median 4.9 (95%CI 2.0–N/A, *n* = 54) months (*χ*^2^ = 21.4, *P* < 0.0001). (B) The benefit of rituximab remained significant when the age effect was added into the analysis. In patients with age > 71 and LDH > 577, there was still a significant difference between rituximab and doxorubicin and nonrituximab nondoxorubicin treatments, median 20.0 (95%CI 14.0–N/A, *n* = 14) months versus 2.0 (95%CI 0.5–N/A, *n* = 5) months (*χ*^2^ = 4.7, *P* = 0.0304). LDH, lactate dehydrogenase.

Because the time from treatment to death is two to three times longer than the time from treatment to first recurrence, we asked whether or not the prolonged survival could be influenced by inherent patient characteristics. We performed a MANOVA to determine the effect on survival from potential predictive factors such as age, CNS disease, and LDH, which were found to be prognostically significant in prior univariate analysis (Fig.11). The combination of age and LDH significantly influenced the time from treatment to death (two degrees of freedom, *P* = 0.0345) and the optimal cutoff for age was 71 years and LDH was 577 mg/dL. In patients with LDH >577, treatment with rituxmab and doxorubicin resulted in significant prolongation of time from treatment to death, median not reached (95%CI 24.0–N/A, *n* = 14) months, as compared to nonrituximab nondoxorubicin regimens, median 4.9 (95%CI 2.0–N/A, *n* = 54) months (*χ*^2^ = 21.4, *P* < 0.0001). When the age effect was added into the analysis, as in age >71 and LDH >577, there was still a significant difference between rituximab and doxorubicin and nonrituximab nondoxorubicin treatments, median 20.0 (95%CI 14.0–N/A, *n* = 14) months versus 2.0 (95%CI 0.5–N/A, *n* = 5) months (*χ*^2^ = 4.7, *P* = 0.0304) (Fig.12). Taken together, both regimens probably treat IVL equally well in prognostically favorable patients but rituximab and doxorubicin works better than nonrituximab nondoxorubicin regimens for those with poor prognosis.

### Patterns of recurrence

The pattern of recurrence after treatment is heterogeneous depending on the organ initially affected. Notably, same-organ recurrence was most common with CNS IVL, or 14 of 16 (88%), compared to 5 of 22 (23%) skin, 5 of 10 (50%) BM and spleen, and 1 of 5 (20%) lung IVL's (Fig.3B). But the small number of patients does not offer enough power for statistical analysis.

## Discussion

IVL is a rare disease with polymorphic presentation that precludes a prospective examination of its natural history. Meta-analysis of existing literature is the only means of obtaining a global view of this disease. Although meta-analysis is often subjected to publication bias, this literature is unique because it comprised primarily clinicopathological description of single cases and small series. These cases still contain important patient data that can be analyzed. Moreover, only three large series have been published to date, all of which were retrospective analyses consisting of predominantly non-CNS IVL's involving the BM, liver, spleen, and skin 7–10. More importantly, the findings in these cases may not be generalized to a broader population because two of the larger series, with 96 and 109 patients, were drawn from a Japanese population 8–10 while a third had 38 cases from an Italian population 7. Therefore, we performed this meta-analysis to elucidate the survival characteristics and prognostic factors in patients with IVL.

Our result indicates that CNS and skin are the most common organs where IVL was found and our findings are consistent with reports documenting IVL's from the European and North American populations 7. However, in the Japanese population, Murase et al. reported 2% and Shimada et al. reported 25% incidence of CNS involvement at initial diagnosis 8–10. Therefore, Asian and non-Asian patients may have different genetic and/or epigenetic influence on the presentation of IVL. Furthermore, CNS IVL has a particularly poor survival rate when compared to non-CNS and skin IVL's. This is most likely because accurate and timely diagnosis of CNS IVL is especially difficult when it is often presented in the elderly with stroke-like symptoms. MRI is often of limited use because the IVL does not cause gadolinium enhancement and a majority of patients’ diagnoses were made postmortem. Indeed, our analysis demonstrated that 60% of CNS IVL cases had the diagnosis established postmortem, sixfold higher than IVL's from other organs. This most likely explains why the time from initial presentation of symptoms to death was significantly shorter than time from diagnosis to death as shown in Figure5 because most of the patients in the first group were diagnosed at postmortem. However, among the few patients who had a timely diagnosis, systemic chemotherapy is efficacious and can prevent further neurological deterioration.

Analysis of clinical and laboratory parameters revealed that age, CNS involvement, and LDH are significant prognostic factors. Notably, age ≥70 and LDH ≥700 IU/L correlated with a shortened survival. Whether or not increasing age corresponds to a greater number and more severe comorbidities remains unclear. Therefore, appropriate clinical decision making may still require oncologists to view the age of IVL patients together with their preexisting comorbidities. Furthermore, LDH is an important prognostic factor for aggressive nodal lymphomas, but it dicotomizes at 500 IU/L with respect to patient survival 11. Our patient's LDH dichotomized at a substantially higher level of 700 IU/L, which corresponds to more than three times the upper limit of normal (∼200 IU/L). Because of the microscopic and intravascular nature of this malignancy, it is plausible that large tumor burden exists in patients without overt radiological findings. Alternatively, turnover of tumor cells and spillage of all intracellular content into the circulation may result in a significantly elevated level of serum LDH than nodal lymphomas where a large fraction of LDH remains sequestered within lymph nodes.

IVL is sensitive to systemic chemotherapy. Notably, the addition of rituximab more than doubled, and the combination of rituximab and doxorubicin more than tripled, the median survival of patients in our analysis. In a retrospective study, Shimada et al.^9^ found an increased response rate, progression-free survival, and overall survival when rituximab was added to conventional chemotherapy. Our MANOVA analysis led us to determine that the greatest benefit of rituximab and doxorubicin treatment, compared to nonrituximab, nondoxorubicin regimens, is found in prognostically unfavorable patients with age >71 and LDH >577. This is probably because both types of regimens have similar efficacy in younger patients with less tumor burden. However, in those with older age and heavier tumor burden, the benefit from rituximab and doxorubicin is magnified when compared to nonrituximab, nondoxorubicin regimens. Younger patients may also have a more intact immune system to counteract the patient's IVL but older patients may need the antibody-mediated complement-dependent cytotoxicity and antibody-dependent cellular cytotoxicity to contain the IVL 12–15. Therefore, it would be important to consider these age- and LDH-related predictive factors in future clinical trials.

The pattern of disease recurrence after treatment also deserves mention. Same-organ recurrence was observed at a higher rate in CNS IVL than IVL's in other organs. This pattern of recurrence may point to a potential difference in the intrinsic biology of CNS versus non-CNS IVL. CNS IVL may have a special predilection for the endothelium of cerebral vasculature and therefore more localized in the brain while IVL's in other organs may herald additional disease in other sites relating to a more diffuse pattern of systemic involvement. Other than the generalized absence of adhesion molecules CD11b and CD18 16, as well as the specific loss of CD29 and CD54 in CNS IVL 2, very little is known about the molecular determinants of this malignancy. A few case reports described the presence of CXCL9 and CXCR3 17, as well as CXCL12 and CXCR4 18, on lymphoma cells and these CXC chemokines may serve as positive regulators for the lymphoma cells to aggregate in specific intravascular space. Vieites et al. reported t(14;18) together with immunohistochemical expression of bcl-6 protein, suggesting germinal center origin of a patient's IVL 19. Others have reported t(11;14)^20^ and t(14;19) 21 but the significance of these translocations remains unclear. Furthermore, search for Epstein–Barr virus genome and membrane proteins were unrevealing 22 but positive immunohistochemical staining of envelope protein from human herpes virus 8 has been reported 23. Future studies using comparative gene profiling of various types of IVL's may shed light on the molecular homing mechanisms to specific organs.

There are a number of formidable barriers that interferes with chemotherapy treatments in patients with CNS IVL. First, timely diagnosis is prevented by the lack of MRI findings and the neurological presentations are often mistaken for stroke. Although serum LDH can be elevated, it is a generalized biomarker of cellular injury and certainly not specific for IVL. Therefore, more specific individual or combinatorial biomarkers from the serum may need to be identified for this disease. Second, the risk of parenchymal brain invasion by CNS IVL remains unknown. To effectively address this question, it will require the prospective neuroimaging of recurrence pattern in patient series as well as careful examination of their brains at autopsy. This issue has treatment implication because if there was a significant risk of parenchymal brain invasion by CNS IVL, high-dose methotrexate in combination with rituximab and doxorubicin may be necessary. Lastly, the dose–response relationship of methotrexate in this disease remains unknown. Only 32 (4%) of the cases we examined used methotrexate. If the risk of parenchymal brain invasion is low, a regimen utilizing a reduced dose of methotrexate may suffice in an effort to prevent drug-induced CNS and non-CNS toxicities in this population of mostly elderly patients.

There are a number of limitations in our study. First, unlike prospective series, the published cases did not have uniform reporting criteria on patient characteristics. Nevertheless, most of the cases included in our analysis contained an extensive description of the patient's illness, disease management, and autopsy findings, which allow us to tabulate them and to perform statistical analysis in aggregate. Second, some of the known prognostic factors for non-Hodgkin's lymphoma, such as the international prognostic index (IPI), revised IPI, and the extent of retroperitoneal disease, cannot be adequately evaluated in our data 14,24. This is because our cases spanned over several decades and some of these prognostic factors had not been developed or recognized. However, LDH was included in a large number of cases and, consistent with published data, our analysis showed that high LDH correlated with poor patient survival. Lastly, patients with CNS IVL die early and nearly half of the patients had their diagnosis established at postmortem. These early deaths preclude an adequate analysis of prognostic factors in this population. Nonetheless, our data showed that a majority of IVL's occur in the CNS and that CNS IVL has the worst prognosis. In addition, older age and higher LDH are important prognostic factors and treatment with rituximab-based chemotherapy regimens offers the best outcome. CNS IVL also recurs primarily in the CNS compared to other IVL's in which same-organ recurrence is less common.

## Supporting Information

Table S1A A list of published literature from 1962 to 2011 on intravascular lymphomatosis.

Table S1B Bibliography for Table S1A.
