# Supplementary material for: The natural history of intravascular lymphomatosis
Source: Cancer Med. 2014 Jun 14;3(4):1010–24. doi: 10.1002/cam4.269 (PMC4303169; doi:10.1002/cam4.269)
Supplement: Table S1A — A list of published literature from 1962 to 2011 on intravascular lymphomatosis. [file cam40003-1010-sd1.docx]

**Supplemental Table 1B.** Bibliography for supplemental table 1A.

1. Abraskia S, Kumar, PD, Kasal J. Two unusual lymphomas/ Case 1: primary malignant lymphoma (diffuse large B-cell lymphoma) of the spleen mimicking splenic abscess. *J Clin Oncol*. 2000;18:3731-3733.
2. Adam DN, Beleznay KM, Randhawa RS, Randhawa RS, Marton M, Zhou Y. Review of intravascular lymphoma with a report of treatment with allogenic peripheral blood stem cell transplant. *Cutis*. 2008;82:267-272.
3. Agar JW, Gates PC, Vaughan SL, Vaughan SL, Machet D. Renal biopsy in angiotropic large cell lymphoma. *Am J Kidney Dis*. 1994;24:92-96.
4. Al-Chalabi A, Sivakumaran M, Holton J, West KP, Wood JK, Abbott RJ. A case of intravascular malignant lymphomatosis (angiotropic lymphoma) with raised perinuclear antineutrophil cytoplasmic antibody titres--a hitherto unreported association. *Clin Lab Haematol.* 1994;6:363-369.
5. Al-Hazzaa SA, Green WR, Mann RB. Uveal involvement in systemic angiotropic large cell lymphoma Microscopic and immunohistochemical studies. Ophthalmology 1993;100:961-965.
6. Al-Humaidan H, Ali A, Ezzat A. Intravascular large-cell lymphoma: Report of an unusual case. *Ann Saudi Med.* 1998;18:553-555.
7. Al-Izzi MS, Sidhu PS, Garside PJ, Menai-Williams R. [Angiotropic large cell lymphoma (angioendotheliomatosis) presenting with protein-losing enteropathy.](http://www.ncbi.nlm.nih.gov/pubmed/3186577) *Postgrad Med.* 1988;64:313-314.
8. Albrecht R, Krebs B, Reusche E, Nadel M, Lencer R, Kretzschmar HA. Signs of rapidly progressive dementia in a case of intravascular lymphomatosis. *Eur Arch Psychiatry Clin Neurosci*. 2005;255:232-235.
9. Amagasaki K, Yamazaki H, Ohmori K, Koizumi H, Hashizume K, Sasaguchi N. Malignant intravascular lymphomatosis associated with venous stenosis. *J Neurosurg.* 1999;90:355-358.
10. Anda T, Haraguchi W, Miyazato H, et al. Ruptured distal middle cerebral artery aneurysm filled with tumor cells in a patient with intravascular large B-cell lymphoma. *J Neurosurg.* 2008;109:492-496.
11. Anghel G, Petrinato G, Severino A, et al. Intravascular B-cell lymphoma: report of two cases with different clinical presentation but rapid central nervous system involvement. *Leuk Lymphoma.* 2003;44:1353-1359.
12. Angioi K, Bodaghi B, Kaminsky P, Mokhtari K, Lubetzki C, LeHoang P. Intravascular lymphoma mimicking a Vogt-Koyanagi-Harada disease. *Ocul Immunol Inflamm*. 2011;19:132-134.
13. Ansbacher L, Low N, Beck D, Boarini F, Jacoby C, Cancilla PA. Neoplastic angioendotheliosis: a clinicopathological entity with multifocal presentation. Case report. *J Neurosurg*. 1981;54:412-415.
14. Ansell J, Bhawan J, Cohen S, Sullivan J, Sherman D. Histiocytic lymphoma and malignant angioendotheliomatosis. One disease or two? *Cancer.* 1982;50:1506-1512.
15. Aoki A, Okamura M, Ueda A, et al .An autopsy case of intravascular lymphomatosis with dermatomyositis. *Intern Med.* 2002;41:241-244.
16. Aoki Y, Takamiya M, Satoh T, Fujita S, Kato H, Maeno Y. A fatal case of hemoperitoneum after ultrasound-guided liver biopsy in a patient with intravascular large B-cell lymphoma. *Leg Med (Tokyo)*. 2011;13:191-195.
17. Aouba A, Diop S, Saadoun D, et al. Severe pulmonary arterial hypertension as initial manifestation of intravascular lymphoma: case report. *Am J Hematol.* 2005;79:46-49.
18. Arboix A, Costa I, Besses C, Sans-Sabrafen. Acute pseudobulbar palsy as the initial presentation of intravascular lymphomatosis. *Eur J Intern Med.* 2004;15:128-130.
19. Arnn ET, Yam LT, Li CY. [Systemic angioendotheliomatosis presenting with hemolytic anemia.](http://www.ncbi.nlm.nih.gov/pubmed/6192702) Am *J Clin Pathol*. 1983;80:246-251.
20. Asagoe K, Fujimoto W, Yoshino T, et al. Intravascular lymphomatosis of the skin as a manifestation of recurrent B-cell lymphoma. *J Am Acad Dermatol.* 2003;48:S1-S4.
21. Askarian F, Xu D. Adrenal enlargement and insufficiency: a common presentation of intravascular large B-cell lymphoma. *Am J Hematol.* 2006;81:411-413.
22. Au WY, Shek WH, Nicholls J, Tse KM, Todd D, Kwong YL. T-cell intravascular lymphomatosis (angiotropic large cell lymphoma): association with Epstein-Barr viral infection. *Histopathology.* 1997;31:563-567.
23. Axelsen RA, Laird PP, Horn M. Intravascular large cell lymphoma: diagnosis on renal biopsy. *Pathology.* 1991;23:241-243.
24. Aznar AO, Montero MA, Rovira R, Vidal FR. Intravascular large B-cell lymphoma presenting with neurological syndromes: clinicopathologic study. *Clin Neuropathol*. 2007;26:180-186.
25. Baehring JM, Longtine J, Hochberg FH. A new approach to the diagnosis and treatment of intravascular lymphoma. *J Neurooncol.* 2003;61:237-248.
26. Bai X, Li X, Wan L, Wang G, Jia N, Geng J. Intravascular large B-cell lymphoma of the kidney: a case report. *Diagn Pathol.* 2011;6:86.
27. Balkema C, Meersseman W, Hermans G, et al. Usefulness of FDG-PET to diagnose intravascular lymphoma with encephalopathy and renal involvement. *Acta Clin Belg.* 2008;63:185-189.
28. Banerjee SS, Harris M. Angiotropic lymphoma presenting in the prostate. *Histopathology.* 1988;12:667-670.
29. Baraniskin A, Jahnert A, Ahle G, et al. Intravascular large B-cell lymphoma presenting as dementia and hemolytic anemia. *Onkologie.* 2010;33:174-177.
30. Barnett CR, Seo S, Husain S, Grossman ME. Intravascular B-cell lymphoma: the role of skin biopsy. *Am J Dermatopathol.* 2008;30:295-299.
31. Bauer A, Perras B, Sufke S, Horny H, Kreft B. Myocardial infarction as an uncommon clinical manifestation of intravascular large cell lymphoma. *Acta Cardiol.* 2005;60:551-555.
32. Baum CL, Stone MS, Liu V. Atypical intravascular CD30+ T-cell proliferation following trauma in a healthy 17-year-old male: first reported case of a potential diagnostic pitfall and literature review. *J Cutan Pathol.* 2009;36:350-354.
33. Baumann TP, Hurwitz N, Karamitopolou-Diamantis E, Probst Am Herrmann R, Steck AJ. Diagnosis and treatment of intravascular lymphomatosis. *Arch Neurol.* 2000;57:374-377.
34. Bazhenova L, Higginbottom P, Mason J. Intravascular lymphoma: a role for single-agent rituximab. *Leuk Lymphoma.* 2006;47:337-341.
35. Ben-Ezra J, Sheibani K, Kendrick FE, Winberg CD, Rappaport H. Angiotropic large cell lymphoma of the prostate gland: an immunohistochemical study. *Hum Pathol.* 1986;17:964-967.
36. Berger JR, Jones R, Wilson D. [Intravascular lymphomatosis presenting with sudden hearing loss.](http://www.ncbi.nlm.nih.gov/pubmed/15850590) *J Neurol Sci.* 2005;232:105-109.
37. Berger TG, Dawson NA. Angioendotheliomatosis. *J Am Acad Dermatol.* 1988;18:407-412.
38. Bergmann M, Terzija-Wessel U, Blasius S, et al. Intravascular lymphomatosis of the CNS: clinicopathologic study and search for expression of oncoproteins and Epstein-Barr virus. *Clin Neurol Neurosurg*. 1994;96:236-243.
39. Beristain X, Azzarelli B. The neurological masquerade of intravascular lymphomatosis. *Arch Neurol*. 2002;59:439-443.
40. Bhagwati NS, Oiseth SJ, Abebe LS, Wiernik PH. Intravascular lymphoma associated with hemophagocytic syndrome: a rare but aggressive clinical entity. *Ann Hematol.* 2004;83:247-250.
41. Bhawan J, Wolff SM, Ucci AA, Bhan AK. Malignant lymphoma and malignant angioendotheliomatosis: one disease. *Cancer.* 1985;55:570-576.
42. Bogomolski-Yahalom V, Lossos IS, Okun E, Sherman Y, Lossos A, Polliack A. Intravascular lymphomatosis--an indolent or aggressive entity? *Leuk Lymphoma.* 1998;29:585-593.
43. Boslooper K, Dijkhuizen D, van der Velden AW, Dal M, Meilof JF, Hoogenberg K. Intravascular lymphoma as an unusual cause of multifocal cerebral infarctions discovered on FDG-PET/CT. *Neth J Med.* 2010;68:261-264.
44. Bots GT. Angioendotheliomatosis of the central nervous system. *Acta Neuropathol.* 1974;28:75-78.
45. Bouzani M, Karmiris T, Rontogianni D, et al. Disseminated intravascular B-cell lymphoma: clinicopathological features and outcome of three cases treated with anthracycline-based immunochemotherapy. *Oncologist.* 2006;11:923-928.
46. Bozzoli V, Tisi MC, D'Alo F, et al. Intravascular large B cell lymphoma: when lymphoma is suspected but routine diagnostic work-up is negative. *Leuk Lymphoma.* 2009;50:1900-1903.
47. Byard RW, Orizaga M. Neoplastic angioendotheliomatosis involving the central nervous system [letter]. *Can J Neurol Sci.* 1986;13:348.
48. Calamia KT, Miller A, Shuster EA, Perniciaro C, Menke DM. Intravascular lymphomatosis. A report of ten patients with central nervous system involvement and a review of the disease process. *Adv Exp Med Biol.* 1999;455:249-265.
49. Carroll TJ, Schelper RL, Goeken JA, Kemp JD. Neoplastic angioendotheliomatosis: immunopathologic and morphologic evidence for intravascular malignant lymphomatosis. *Am J Clin Pathol.* 1986;85:169-175.
50. Carter DK, Batts KP, de Groen PC, Kurtin PJ. Angiotropic large cell lymphoma (intravascular lymphomatosis) occurring after follicular small cleaved cell lymphoma. *Mayo Clin Proc.* 1996;71:869-873.
51. Cerroni L, Massone C, Kutzner H, Mentzel T, Umbert P, Keri H. Intravascular large T-cell or NK-cell lymphoma: a rare variant of intravascular large cell lymphoma with frequent cytotoxic phenotype and association with Epstein-Barr virus infection. *Am J Surg Pathol.* 2008;32:891-898.
52. Cerroni L, Zalaudek I, Kerl H. Intravascular large B-cell lymphoma colonizing cutaneous hemangiomas. *Dermatology.* 2004 ;209:132-134.
53. Chakravarty K, Goyal M, Scott DG, McCann BG. Malignant 'angioendotheliomatosis'--(intravascular lymphomatosis) an unusual cutaneous lymphoma in rheumatoid arthritis. *Br J Rheumatol.* 1993;32:932-934.
54. Chang A, Zic JA, Boyd AS. Intravascular large cell lymphoma: a patient with asymptomatic purpuric patches and a chronic clinical course. *J Am Acad Dermatol.* 1998;39:318-321.
55. Chapin JE, Davis LE, Kornfeld M, Mandler RN. Neurologic manifestations of intravascular lymphomatosis. *Acta Neurol Scand.* 1995;91:494-499.
56. Chapman JE, Loy WA, Chapman JE Jr. Cerebral angioendotheliomatosis associated with hemispheric symptoms of carotid arterial origin. *J Vasc Surg*. 1985;2:281-284.
57. Chaukiyal P, Singh S, Woodlock T, Dolan JG, Bruner K. Intravascular large B-cell lymphoma with multisystem involvement. *Leuk Lymphoma.* 2006;47:1688-1690.
58. Chen TM, Crow MK, Teller C. Angiotropic large-cell lymphoma with striking blue plaques. *J Am Acad Dermatol.* 2003;48:633-634.
59. Chen P, Gaetjens E, Sher J, et al: [Malignant angioendotheliomatosis manifesting as ascending spinal cord dysfunction.](http://www.ncbi.nlm.nih.gov/pubmed/3477718) *N Y State J Med.* 1987;87:470-472.
60. Chen M, Qiu B, Kong J, Chen J. Angiotropic T cell lymphoma. *Chin Med J (Engl)*. 1998;111:762-764.
61. Chim CS, Choy C, Ooi GC, Chung LP, Wong KK, Liang R. Two unusual lymphomas. Case 2: pulmonary intravascular lymphomatosis. *J Clin Oncol.* 2000;18:3733-3735.
62. Chim CS, Loong F. Intravascular lymphomatosis of the prostate gland. *Br J Haematol.* 2002;119:2
63. Chinen Y, Nakao M, Sugitani-Yamamoto M, et al: Intravascular B-cell lymphoma with hypercalcemia as the initial presentation. *Int J Hematol.* 2011;94:567-570.
64. Cho K, Kim C, Yang S, Kim B, Kim J. Angiocentric T cell lymphoma of the skin presenting as inflammatory nodules of the leg. *Clin Exp Dermatol*. 1997;22:104-108.
65. Chu P, Costa J, Lachman MF. Angiotropic large cell lymphoma presenting as adrenal insufficiency. *Hum Pathol.* 1996;27:209-211.
66. Clark WC, Dohan FC, Moss T, Schweitzer JB. Immunocytochemical evidence of lymphocytic derivation of neoplastic cells in malignant angioendotheliomatosis. *J Neurosurg.* 74:757-62, 1991
67. Collins KA, Davis GJ. Angiotropic large cell lymphoma. *South Med J*. 1995;88:235-238.
68. Conlin PA, Orden MB, Hough TR, Morgan DL. Myeloperoxidase-positive intravascular large B-cell lymphoma. *Arch Pathol Lab Med.* 2001;125:948-950.
69. Csomor J, Kaszas I, Kollar B, et al: Prolonged survival using anti-CD20 combined chemotherapy in primary prostatic intravascular large B-cell lymphoma. *Pathol Oncol Res.* 2008;14:281-284.
70. Curtis JL, Warnock ML, Conrad DJ, Helfend LK, Boushey HA. Intravascular (angiotropic) large-cell lymphoma ('malignant angioendotheliomatosis') with small vessel pulmonary vascular obstruction and hypercalcemia. *West J Med*. 1991;155:72-76.
71. D'Agati V, Sablay LB, Knowles DM, Walter L. Angiotropic large cell lymphoma (intravascular malignant lymphomatosis) of the kidney: presentation as minimal change disease. *Hum Pathol.* 1989;20:263-268.
72. Daniel SE, Rudge P, Scaravilli F. Malignant angioendotheliosis involving the nervous system: support for a lymphoid origin of the neoplastic cells. *J Neurol Neurosurg Psychiatry*. 1987;50:1173-1177.
73. Davis TS. Intravascular lymphoma presenting with cauda equina syndrome: treated with CHOP and rituxan. *Leuk Lymphoma.* 2003;44:887-888.
74. Debiais S, Bonnaud I, Cottier JP, et al. A spinal cord intravascular lymphomatosis with exceptionally good outcome. *Neurology.* 2004;63:1329-1330.
75. Dĕdic K, Belada D, Zák P, Nozicka Z. Intravascular large B-cell lymphoma presenting as cutaneous panniculitis. *Acta Medica (Hradec Kralove).* 2003;46:121-123.
76. Deisch J, Fuda FB, Chen W, et al: Segmental tandem triplication of the MLL gene in an intravascular large B-cell lymphoma with multisystem involvement: a comprehensive morphologic, immunophenotypic, cytogenetic, and molecular cytogenetic antemortem study. *Arch Pathol Lab Med.* 2009;133:1477-1482.
77. Demirer T, Dail DH, Aboulafia DM. [Four varied cases of intravascular lymphomatosis and a literature review.](http://www.ncbi.nlm.nih.gov/pubmed/8156502) *Cancer.* 1994;73:1738-1745.
78. Deschamps L, Signate A, Delaunay C, et al. A blind skin biopsy diagnosing an intravascular large B-cell lymphoma. *Eur J Dermatol.* 2011;21:114-115.
79. Deusch E, Mayr A, Hobisch-Hagen P, et al. Angiotropic large B-cell lymphoma misdiagnosed as urosepsis with multiple organ dysfunction syndrome. *Acta Anaesthesiol Scand.* 1999;43:100-103.
80. Devlin T, Moll S, Hulette C, Morganlander JC. Intravascular malignant lymphomatosis with neurologic presentation: factors facilitating antemortem diagnosis. *South Med J.* 1998;91:672-676.
81. DiGiuseppe JA, Nelson WG, Seifter EJ, Boitnott JK, Mann RB. Intravascular lymphomatosis: a clinicopathologic study of 10 cases and assessment of response to chemotherapy. *J Clin Oncol.* 1994;12:2573-2579.
82. Dominguez FE, Rosen LB, Kramer HC. Malignant angioendotheliomatosis proliferans. Report of an autopsied case studied with immunoperoxidase. *Am J Dermatopathol.* 1986;8:419-425.
83. Domizio P, Hall PA, Cotter F, et al: Angiotropic large cell lymphoma (ALCL): morphological, immunohistochemical and genotypic studies with analysis of previous reports. *Hematol Oncol.* 1989;7:195-206.
84. Dozić S, Suvaković V, Cvetković D, Jevtovic D, Skender M. Neoplastic angioendotheliomatosis (NAE) of the CNS in a patient with AIDS subacute encephalitis, diffuse leukoencephalopathy and meningo-cerebral cryptococcosis. *Clin Neuropathol*. 1990;9:284-289.
85. Drlicek M, Grisold W, Liszka U, Hitzenberger P, Machacek E. Angiotropic lymphoma (malignant angioendotheliomatosis) presenting with rapidly progressive dementia. *Acta Neuropathol*. 1991;82:533-555.
86. Drobacheff C, Blanc D, Zultak M, et al: Malignant angioendotheliomatosis. Reclassification as an angiotropic lymphoma. *Int J Dermatol*. 1989;28:454-456.
87. Duan X, Lapus A, Brown RE, Chen L. Intravascular Large B-cell Lymphoma Presenting as Cholecystitis and Pancytopenia: Case Report with Literature Review. *Ann Clin Lab Sci.* 41:262-6, 2011
88. Dubas F, Saint-Andre JP, Pouplard-Barthelaix A, Delestre F, Emile J. Intravascular malignant lymphomatosis (so-called malignant angioendotheliomatosis): a case confined to the lumbosacral spinal cord and nerve roots*. Clin Neuropathol.* 1990;9:115-120.
89. Dufau JP, Le Tourneau A, Molina T, et al. Intravascular large B-cell lymphoma with bone marrow involvement at presentation and haemophagocytic syndrome: two Western cases in favour of a specific variant. *Histopathology.* 2000;37:509-512.
90. Dunphy CH. Primary cutaneous angiotropic large-cell lymphoma in a patient with acquired immunodeficiency syndrome. *Arch Pathol Lab Med.* 1995;119:757-759.
91. Elner VM, Hidayat AA, Charles NC, et al. Neoplastic angioendotheliomatosis. A variant of malignant lymphoma immunohistochemical and ultrastructural observations of three cases. *Ophthalmology.* 1986;93:1237-1245.
92. Emura I, Naito M, Wakabayashi M, Yoshizawa H, Arakawa M, Chou T. Detection of circulating tumor cells in a patient with intravascular lymphomatosis: a case study examined by the cytology method. *Pathol Int.* 1998;48:63-66.
93. Estalilla OC, Koo CH, Brynes RK, Medeiros LJ. Intravascular large B-cell lymphoma. A report of five cases initially diagnosed by bone marrow biopsy. *Am J Clin Pathol*. 1999;112:248-255.
94. Evert M, Lehringer-Polzin M, Möbius W, Pfeifer U. Angiotropic large-cell lymphoma presenting as pulmonary small vessel occlusive disease. *Hum Pathol.* 2000;31:879-882.
95. Feldmann R, Schierl M, Sittenthaler M, et al. Intravascular large B-cell lymphoma of the skin: typical clinicalmanifestations and a favourable response to rituximab-containing therapy. *Dermatology.* 2009;219:344-346.
96. Felizardo M, Mendes AC, Fernandes A, et al. Intravascular pulmonary lymphoma with good response to treatment A case report. *Rev Port Pneumol.* 2008;14:857-868.
97. Ferry JA, Harris NL, Picker LJ, et al. Intravascular lymphomatosis (malignant angioendotheliomatosis). A B-cell neoplasm expressing surface homing receptors. *Mod Pathol.* 1988;1:444-452.
98. Ferry JA, Sohani AR, Longtine JA, Schwartz RA, Harris NL. HHV8-positive, EBV-positive Hodgkin lymphoma-like large B-cell lymphoma and HHV8-positive intravascular large B-cell lymphoma. *Mod Pathol.* 2009;22:618-626.
99. Fiegl M, Greil R, Pechlaner C, Krugmann J, Dirnhofer S. Intravascular large B-cell lymphoma with a fulminant clinical course: a case report with definite diagnosis post mortem. *Ann Oncol.* 2002;13:1503-1506.
100. Fievez M, Fievez C, Hustin J. [Proliferating systematized angioendotheliomatosis.](http://www.ncbi.nlm.nih.gov/pubmed/5096839) Arch Dermatol 1971;104:320-324.
101. Flores-Vázquez F, de León-Bojorge B, Ortiz-Hidalgo C, Capurso M. Intravascular lymphoma presenting with clinical features of cholecystitis. *South Med J.* 2001;94:946-947.
102. Fozza C, Bonfigli S, Conti M, Dore F, Longinotti M. Long-lasting fever of unknown origin preceding the diagnosis of intravascular lymphomatosis: a further case stimulates some remarks. *Am J Hematol.* 2003;74:211-213.
103. Fujiwara A, Nagayama S, Amada S, Shimamoto T, Shimao Y, Hayashi T. Intravascular large B-cell lymphoma involving mainly the uterus: report of a case using liquid-based cytology of the endometrium. *Acta Cytol*. 2010;54:787-792.
104. Fukuchi M, Fushimi S, Yoneya M, Hirayama A. An autopsy case of intravascular malignant lymphoma presenting with intracranial B-cell type malignant lymphoma. *Noshuyo Byori.* 1996;13:119-125.
105. Fukushima A, Okada Y, Tanikawa T, et al. Primary bilateral adrenal intravascular large B-cell lymphoma associated with adrenal failure. *Intern Med.* 2003;42:609-614.
106. Fulling KH, Gersell DJ. Neoplastic angioendotheliomatosis. Histologic, immunohistochemical, and ultrastructural findings in two cases. *Cancer.* 1983;51:1107-1118.
107. Gabor EP, Sherwood T, Mercola KE. Intravascular lymphomatosis presenting as adult respiratory distress syndrome. *Am J Hematol.* 1997;56:155-160.
108. Ganeshan A, Soonawalla Z, De M, Baxter J. Intravascular lymphoma: a diagnostic enigma. *J R Soc Med*. 2002;95:37-38.
109. Garg A, Hosfield EM, Brickner L. Disseminated intravascular large B cell lymphoma with slowly decreasing high-density lipoprotein cholesterol. *South Med J.* 2011;104:53-56.
110. Gaul C, Hanisch F, Neureiter D, Bhermann C, Nenundorfer B, Winterholler M. Intravascular lymphomatosis mimicking disseminated encephalomyelitis and encephalomyelopathy. *Clin Neurol Neurosurg.* 2006;108:486-489.
111. Georgin-Lavialle S, Darmon M, Galicier L, Fysekidis M, Azoulay E. Intravascular lymphoma presenting as a specific pulmonary embolism and acute respiratory failure: a case report. *J Med Case Reports*. 2009;12:7253.
112. Ghorbani RP, Shokouh-Amiri H, Gaber L. Intragraft angiotropic large-cell lymphoma of T cell-type in a long-term renal allograft recipient. *Mod Pathol*. 9:671, 1996.
113. Gill S, Melosky B, Haley L, ChanYan C. Use of random skin biopsy to diagnose intravascular lymphoma presenting as fever of unknown origin. *Am J Med*. 2003;114:56-58.
114. Gioulis M, Ben G, Iuzzolino P, De Biasi F, Marchini C, Zambito Marsala S. Subacute cognitive disorders as initial presentation of intravascular lymphoma: a case report and review of literature. *Neurol Sci.* 2010;31:369-372.
115. Glass J, Hochberg FH, Miller DC. Intravascular lymphomatosis. A systemic disease with neurologic manifestations. *Cancer.* 1993;71:3156-3164.
116. Gleason BC, Brinster NK, Granter SR, Pinkus GS, Lindeman NI, Miller DM. Intravascular cytotoxic T-cell lymphoma: A case report and review of the literature. *J Am Acad Dermatol.* 2008;58:290-294.
117. Go A, Venugopal P, Loew J, Djordjevic D. Durable remission of intravascular lymphoma with central nervous system involvement following chemotherapy and rituximab. *Clin Adv Hematol Oncol*. 2006;4:439-441.
118. Goh SG, Chuah KL, Tan PH. [Intravascular lymphomatosis of the lung and liver following eyelid lymphoma in a Chinese man and review of primary pulmonary intravascular lymphomatosis.](http://www.ncbi.nlm.nih.gov/pubmed/11902454) *Pathology.* 2002;34:82-85.
119. Grove CS, Robbins PD, Kermode AG. Intravascular lymphoma presenting as progressive paraparesis. *J Clin Neurosci.* 2008;15:1056-1058.
120. Gupta AK, Lipa M, Haberman HF. [Proliferating angioendotheliomatosis. Case with long survival and review of literature.](http://www.ncbi.nlm.nih.gov/pubmed/3513710) *Arch Dermatol.* 1986;122:314-319.
121. Haber H, Harris-Jones JN, Wells AL. Intravascular Endothelioma (Endothelioma IN-SITU, Systemic Endotheliomatosis). *J Clin Pathol.* 1964;17:608-611.
122. Hadjileontis CG, Kostopoulos IS, Kaloutsi VD, Nikolaou AC, Kotloula VA, Papadimitriou CS. An extremely rare case of synchronous occurrence in the larynx of intravascular lymphoma and in situ squamous cell carcinoma. *Leuk Lymphoma.* 2003;44:1053-1057.
123. Hamada K, Hamada T, Satoh M, et al. Two cases of neoplastic angioendotheliomatosis presenting with myelopathy. *Neurology.* 1991;41:1139-1140.
124. Han K, Haley JC, Carlson K, Pinter-Brown L, Soriano T. Regression of cutaneous intravascular lymphoma with rituximab. *Cutis.* 2003;72:137-140.
125. Hanihara T, Takahashi T, Shimada T, Mizuguchi M, Yagishita S. Parathyroid hormone-related protein-associated hypercalcemia in probable intravascular lymphoma of B-cell type. *Am J Hematol.* 1996;53:144-145.
126. Harris CP, Sigman JD, Jaeckle KA. Intravascular malignant lymphomatosis: amelioration of neurological symptoms with plasmapheresis. *Ann Neurol.* 1994;35:357-359.
127. Hayashi T, Watanabe E, Ogawa M, et al. Angiotropic B-cell lymphoma presenting with progressive dementia: an autopsy case and review of the literature in Japan. *Intern Med.* 1995;34:1134-1139.
128. Heafield MT, Carey M, Williams AC, Cullen M. Neoplastic angioendotheliomatosis: a treatable "vascular dementia" occurring in an immunosuppressed transplant patient. *Clin Neuropathol.* 1993;12:102-106.
129. Heinrich A, Vogelgesang S, Kirsch M, Khaw AV. Intravascular lymphomatosis presenting as rapidly progressive dementia. *Eur Neurol.* 2005;54:55-85.
130. Helm TN, Bergfeld WF, Elston D. Angiotropic lymphoma: malignant angioendotheliomatosis. *Cutis.* 1992;50:204-206.
131. Hishikawa N, Niwa H, Hara T, et al. An autopsy case of lymphomatosis cerebri showing pathological changes of intravascular large B-cell lymphoma in visceral organs. *Neuropathology.* 2011;31:612-619.
132. Hofman MS, Fields P, Yung L, Mikhaeel NG, Ireland R, Nunan T. Meningeal recurrence of intravascular large B-cell lymphoma: early diagnosis with PET-CT. *Br J Haematol.* 2007;137:386.
133. Holmes NE, Gordon CL, Lightfoot N, et al. Intravascular large B cell lymphoma: an elusive cause of pyrexia of unknown origin diagnosed postmortem. *Clin Infect Dis.* 2010;51:e61-64.
134. Holmoy T, Nakstad PH, Fredo HL, Kumar T. Intravascular large B-cell lymphoma presenting as cerebellar and cerebral infarction. *Arch Neu*r*ol.* 2007;64:754-755.
135. Horvath B, Demeter J, Eros N, et al. Intravascular large B-cell lymphoma: remission after rituximab-cyclophosphamide, doxorubicin, vincristine, and prednisolone chemotherapy. *J Am Acad Dermatol.* 2009;61:885-888.
136. Hoshino A, Kawada E, Ukita T, et al. Usefulness of FDG-PET to diagnose intravascular lymphomatosis presenting as fever of unknown origin. *Am J Hematol.* 2004;76:236-239.
137. Hsiao CH, Su IJ, Hsieh SW, et al. Epstein-Barr virus-associated intravascular lymphomatosis within Kaposi's sarcoma in an AIDS patient. *Am J Surg Pathol*. 1999;23:482-487.
138. Hsieh MS, Yeh YC, Chou YH, Lin CW. Intravascular large B cell lymphoma in Taiwan: an Asian variant of non-germinal-center origin. *J Formos Med Assoc*. 2010;109:185-191.
139. Hsu YH, Tseng BY, Shyu WC, et al: Intravascular lymphomatosis mimicking acute disseminated encephalomyelitis: a case report. *Kaohsiung J Med Sci*. 21:93-7, 2005
140. Hundsberger T, Cogliatti S, Kleger GR, et al. Intravascular lymphoma mimicking cerebral stroke: report of two cases. *Case Rep Neurol.* 2011;3:278-283.
141. Hwang WS, Jung CW, Ko YH, Seo SW, Na DL. [Intravascular Lymphomatosis Presenting as Acute Hemispheric Dysfunction.](http://www.ncbi.nlm.nih.gov/pubmed/21454094) *J Stroke Cerebrovasc Dis.* Available from URL: http://www.sciencedirect.com/science/article/pii/S1052305711000462.

1. Iijima M, Fujita A, Uchigata M, Katoo H. Change of brain MRI findings in a patient with intravascular malignant lymphomatosis. *Eur J Neurol*. 2007;14:e4-5.
2. Im SH, Lee SY, Kim NH. Headache as the only presentation of intravascular lymphoma. *Headache.* 2008;48:627-629.
3. Imai H, Kajimoto K, Taniwaki M, et al. Intravascular large B-cell lymphoma presenting with mass lesions in the central nervous system: a report of five cases. *Pathol Int.* 2004;54:231-236.
4. Imamura K, Awaki E, Aoyama Y, et al. Intravascular large B-cell lymphoma following a relapsing stroke with temporary fever: a brain biopsy case. *Intern Med.* 2006;45:693-695.
5. Inooka G, Ishikawa S, Saito T, Saito K, Kamoshida T, Kuzuya T. An autopsy case of intravascular lymphomatosis (neoplastic angioendotheliomatosis) accompanied by high fever, hypertension and without focal sign. *Intern Med.* 1992;31:666-670.
6. Ip M, Chan KW, Chan IK. Systemic inflammatory response syndrome in intravascular lymphomatosis. *Intensive Care Med.* 1997;23:783-786.
7. Ishida M, Hodohara K, Yoshida T, Okabe H. Intravascular large B-cell lymphoma colonizing in senile hemangioma: a case report and proposal of possible diagnostic strategy for intravascular lymphoma. *Pathol Int.* 2011;61:555-557.
8. Ishiguro T, Takayanagi N, Yanagisawa T, et al. Pulmonary microvascular cytology can detect tumor cells of intravascular lymphoma. *Intern Med.* 2009;48:1425-1428.
9. Ishii W, Ito S, Kondo Y, et al. Intravascular large B-cell lymphoma with acute abdomen as a presenting symptom in a patient with systemic lupus erythematosus. *J Clin Oncol.* 2008;26:1553-1555.
10. Ishiko J, Mizuki M, Yasumi M, et al. An indolent subtype of "intravascular lymphoma": A case with a 3-year history of LDH elevation. *Leuk Lymphoma.* 2007;48:1872-1874.
11. Isimbaldi G, Corral L, Songia S, Valentine MG, De Bianchi S, Biondi A. An unusual presentation of a case of T cell angiotropic (intravascular) lymphoma. *Leukemia.* 2000;14:2321-2322.
12. Ito M, Kim Y, Choi JW, Ozawa H, Fujino M. Prevalence of intravascular large B-cell lymphoma with bone marrow involvement at initial presentation. *Int J Hematol*. 2003;77:159-163.
13. Jalkanen S, Aho R, Kallajoki M, et al. Lymphocyte homing receptors and adhesion molecules in intravascular malignant lymphomatosis. *Int J Cancer.* 1989;44:777-782.
14. Jang HJ, Lee KS, Han J. Intravascular lymphomatosis of the lung: radiologic findings. *J Comput Assist Tomogr.* 1998;22:427-429.
15. Jardin F, Callonnec F, Contentin N, et al. Intravascular large B-Cell lymphoma with bone marrow involvement and superior sagittal sinus thrombosis: report of a case successfully treated with a CHOP/rituximab combination regimen. *Clin Lymphoma.* 2005;6:46-49.
16. Jiang QL, Pytel P, Rowin J. Disseminated intravascular large-cell lymphoma with initial presentation mimicking Guillain-Barré syndrome. *Muscle Nerve.* 2010;42:133-136.
17. Jitpratoom P, Yuckpan P, Sitthinamsuwan P, Chotinaiwattarakul W, Chinthammir Y. Progressive multifocal cerebral infarction from intravascular large B cell lymphoma presenting in a man: a case report. *J Med Case Reports.* 2011;5:24.
18. Jones JM, Ceballos R. Neoplastic angioendotheliomatosis and prostatic carcinoma coexisting in a patient. A case report. *Ala J Med Sci.* 1986;23:318-321.
19. Kaku N, Seki M, Doi S, et al. [A case of intravascular large B-cell lymphoma (IVLBCL) with no abnormal findings on chest computed tomography diagnosed by random transbronchial lung biopsy.](http://www.ncbi.nlm.nih.gov.ezp-prod1.hul.harvard.edu/pubmed/21173545) *Intern Med.* 2010;49:2697-2701.
20. Kakumitsu H, Higuchi M, Tanaka K, Shibuya T. Nephrotic syndrome in a patient with intravascular lymphomatosis. *Intern Med.* 2003;42:98-101.
21. Kamath NV, Gilliam AC, Nihal M, Spiro TP, Wood GS. Primary cutaneous large B-cell lymphoma of the leg relapsing as cutaneous intravascular large B-cell lymphoma. *Arch Dermatol.* 2001;137:1657-1658.
22. Kameoka Y, Takahashi N, Komatsuda A, et al. Kidney-limited intravascular large B cell lymphoma: a distinct variant of IVLBCL? *Int J Hematol.* 2009;89:533-537.
23. Kameoka Y, Takahashi N, Tagawa, et al. A case of intravascular large B-cell lymphoma of the cutaneous variant: the first case in Asia. *Int J Hematol.* 2010;91:146-148.
24. Kamesaki H, Matsui Y, Ohno Y, et al. Angiotropic lymphoma with histologic features of neoplastic angioendotheliomatosis presenting with predominant respiratory and hematologic manifestations. *Am J Clin Pathol* 1990;94:768-772.
25. Kanda M, Suzumiya J, Ohshima K, Tamura K, Kikuchi M. Intravascular large cell lymphoma: clinicopathological, immuno-histochemical and molecular genetic studies. *Leuk Lymphoma. 34:569-580.*
26. Kanda M, Suzumiya J, Ohshima K, et al. Analysis of the immunoglobulin heavy chain gene variable region of intravascular large B-cell lymphoma. *V*i*rchows Arch.* 2001;439:540-546.
27. Kanno M, Nakamura S, Kawahara M, et al: Chemotherapy-resistant intravascular lymphoma accompanied by ADAMTS13 inhibitor successfully treated with rituximab. *Int J Hematol.* 88:345-7, 2008
28. Kano M, Yoshida J, Hashizume Y, et al. Factor VIII can be positive in a special type of malignant lymphoma, intravascular malignant lymphomatosis: an immunohistochemical investigation. *Noshuyo Byori.* 1994;11:135-141.
29. Kao NL, Broy S, Tillawi I. Malignant angioendotheliomatosis mimicking systemic necrotizing vasculitis. *J Rheumatol.* 1992;19:1133-1135.
30. Kasuya A, Hashizume H, Takigawa M. Early diagnosis of recurrent diffuse large B-cell lymphoma showing intravascular lymphoma by random skin biopsy. *J Dermatol.* 2011;38:571-574.
31. Katalinic D, Valkovic T, Lucin K, Rudez J. Intravascular lymphoma and thyroid gland. *Coll Antropol*. 2006;30:239-241.
32. Kato M, Ohshima K, Mizuno M, et al. Analysis of CXCL9 and CXCR3 expression in a case of intravascular large B-cell lymphoma. *J Am Acad Dermatol.* 2009;61:888-91.
33. Kauh YC, McFarland JP, Carnabuci GG, Carnabuci CG, Luscombe HA. [Malignant proliferating angioendotheliomatosis.](http://www.ncbi.nlm.nih.gov/pubmed/7396545) *Arch Dermatol.* 1980;116:803-806.
34. Kawahara M, Kanno M, Matsumoto M, Nakamura S, Fuhimura Y, Ueno S. Diffuse neurodeficits in intravascular lymphomatosis with ADAMTS13 inhibitor. *Neurology.* 2004;63:1731-1733.
35. Kawamura T, Sando Y, Tajima S, et al. Pulmonary intravascular lymphoma complicated with Pneumocystis carinii pneumonia: a case report. *Jpn J Clin Oncol.* 2001;31:333-336.
36. Kaya H, Yoshida T. A case of intravascular lymphoma complicated with Fournier's syndrome due to multidrug-resistant Pseudomonas aeruginosa. *J Clin Exp Hematol.* 2011;51:115-118.
37. Kayano H, Katayama I. Primary hepatic lymphoma presenting as intravascular lymphomatosis. *Arch Pathol Lab Med*. 1990;114:580-584.
38. Keahey TM, Guerry D, Tuthill RJ, Bondi EE. [Malignant angioendotheliomatosis proliferans treated with doxorubicin.](http://www.ncbi.nlm.nih.gov/pubmed/7092278) *Arch Dermatol.* 1982;118:512-514.
39. Kenez J, Barsi P, Majtenyi K, et al. Can intravascular lymphomatosis mimic sinus thrombosis? A case report with 8 months' follow-up and fatal outcome. *Neuroradiology.* 2000;42:436-440.
40. Khoury H, Dalal BI, Nantel SH. Intravascular lymphoma presenting with bone marrow involvement and leukemic phase. *Leuk Lymphoma.* 2003;44:1043-1047.
41. Khoury H, Lestou VS, Gascoyne RD, et al. Multicolor karyotyping and clinicopathological analysis of three intravascular lymphoma cases. *Mod Pathol.* 2003;16:716-724.
42. Kidson-Gerber G, Bosco A, Maccallum S, Dunkley S. Two cases of intravascular lymphoma: highlighting the diagnostic difficulties in pyrexia of unknown origin. *Intern Med. J.* 2005;35:569-570.
43. Kinoshita T, Sugihara S, Matusue E, et al. Intravascular malignant lymphomatosis: diffusion-weighted magnetic resonance imaging characteristics. *Acta Radiol.* 2005;46:246-249.
44. Kitanaka A, Kubota Y, Imataki O, et al. Intravascular large B-cell lymphoma with FDG accumulation in the lung lacking CT/(67)gallium scintigraphy abnormality. *Hematol Oncol.* 2009;27:46-49.
45. Kivity S, Shalmon B, Sidi Y. Guillain-Barre syndrome: an unusual presentation of intravascular lymphoma. *Isr Med Assoc J.* 2006;8:137-138.
46. Kiyohara T, Kumakiri M, Kobayashi H, Shimizu T, Ohkawara A, Ohnuki M. A case of intravascular large B-cell lymphoma mimicking erythema nodosum: the importance of multiple skin biopsies. *J Cutan Pathol.* 2000;27:413-418.
47. Ko YH, Han JH, Go JH, et al. [Intravascular lymphomatosis: a clinicopathological study of two cases presenting as an interstitial lung disease.](http://www.ncbi.nlm.nih.gov/pubmed/9447388) *Histopathology.* 1997;31:555-562.
48. Kobayashi T, Ohno H. Intravascular large B-cell lymphoma associated with t(14;19)(q32;q13) translocation. *Intern Med.* 2011;50:2007-2010.
49. Kobrich U, Falk S, Karhoff M, Middeke B, Anselstetter V, Stutte HJ. Primary large cell lymphoma of the splenic sinuses: a variant of angiotropic B-cell lymphoma (neoplastic angioendotheliomatosis)? *Hum Pathol.* 1992;23:1184-1187.
50. Koizumi M, Nishimura M, Yokota A, Munekata S, Kobayashi T, Saito Y. Successful treatment of intravascular malignant lymphomatosis with high-dose chemotherapy and autologous peripheral blood stem cell transplantation. *Bone Marrow Transplant.* 2001;27:1101-1103.
51. Kong YY, Dai B, Sheng WQ, et al. Intravascular large B-cell lymphoma with cutaneous manifestations: a clinicopathologic, immunophenotypic and molecular study of three cases. *J Cutan Pathol*. 2009;36:865-870.
52. Kotake T, Kosugi S, Takimoto T, et al. Intravascular large B-cell lymphoma presenting pulmonary arterial hypertension as an initial manifestation. *Intern Med.* 2010;49:51-54.
53. Koyama T, O'uchi T, Matsue K. [Neurolymphomatosis involving the trigeminal nerve and deep peroneal nerve in a patient with relapsed intravascular large B-cell lymphoma.](http://www.ncbi.nlm.nih.gov/pubmed/20528901) *Eur J Haematol.* 2010;85:275-276.
54. Kraus MD, Jones D, Bartlett NL. Intravascular lymphoma associated with endocrine dysfunction: a report of four cases and a review of the literature. *Am J Med.* 1999;107:169-176.
55. Kreiss Y, Schwartz E, Kaminski N, et al. Unique pulmonary presentation of intravascular large cell lymphoma. *Respir Med*. 1998;92:1087-1089.
56. Krishnan C, Moline S, Anders K, Warnke RA. Intravascular ALK-positive anaplastic large-cell lymphoma mimicking inflammatory breast carcinoma. *J Clin Oncol.* 2009;27:2563-2565.
57. Krokowski M, Sellmann L, Feller AC. Intravascular large B-cell lymphoma within a subcutaneous cavernous haemangioma. *Br J Haematol*. 2010;151:2.
58. Kumar N, Keegan BM, Rodriguez FJ, Hammack JE, Kantarci OH. Intravascular lymphoma presenting as a longitudinally-extensive myelitis: diagnostic challenges and etiologic clues. *J Neurol Sci*. 2011;303:146-149.
59. Kuo TT, Chen MJ, Kuo MC. Cutaneous intravascular NK-cell lymphoma: report of a rare variant associated with Epstein-Barr virus. *Am J Surg Pathol.* 2006;30:1197-1201.
60. Kurrein F. Systemic angioendotheliomatosis with metastases. *J Clin Pathol.* 1976;29:347-353.
61. Kusaba T, Hatta T, Tanda S, et al. Histological analysis on adhesive molecules of renal intravascular large B cell lymphoma treated with CHOP chemotherapy and rituximab. *Clin Nephrol*. 2006;65:222-226.
62. Kuvliev E, Glamour T, Shekar R, West BC. Angiotropic large cell lymphoma presenting as fever of unknown origin. *Am J Med Sci.* 1999;317:266-268.
63. Kuwabara H. Intravascular Lymphomatosis presenting as bilateral adrenal enlargement and insufficiency. *Acta Cytologica.* 1999;43:975-976.
64. Kuwahara K, Fukata J, Kamio M, Mochizuki T, Tsuchiya A, Tanaka S. Angiotropic large cell lymphoma which infiltrated to the adrenal glands presenting as reversible adrenal insufficiency. *Intern Med.* 1998;37:73-76.
65. Lacomis D, Smith TW, Long RR. Angiotropic lymphoma (intravascular large cell lymphoma) presenting with cauda equina syndrome. *Clin Neurol Neurosurg.* 1992;94:311-315.
66. Langan SM, O'Briain S, Barnes L. Dermatomyositis associated with angiotropic lymphoma. *Clin Exp Dermatol.* 2003;28:597-599.
67. Lannoo L, Smets S, Steenkiste E, et al. Intravascular large B-cell lymphoma of the uterus presenting as fever of unknown origin (FUO) and revealed by FDG-PET. *Acta Clin Belg.* 2007;62:187-190.
68. Lapkuviene O, Forchetti D, Roepke JE. Unusual sites of involvement by hematologic malignancies Case 1 Intravascular large B-cell lymphoma presenting with CNS symptoms. *J Clin Oncol.* 2001;19:3988-3991.
69. Laurino L, Melato M. [Malignant angioendotheliomatosis (Angiotropic lymphoma) of the gallbladder.](http://www.ncbi.nlm.nih.gov/pubmed/2117313) *Virchows Arch A Pathol Anat Histopathol.* 1990;417:243-246.
70. Le EN, Gerstenblith MR, Gelber AC, et al. The use of blind skin biopsy in the diagnosis of intravascular B-cell lymphoma. *J Am Acad Dermatol.* 2008;59:148-151.
71. Le K, Lim A, Bullpitt P, Wood G. Intravascular B-cell lymphoma diagnosed by skin biopsy. *Australas J Dermatol.* 2005;46:261-265.
72. Lee BS, Frankfort BJ, Eberhart CG, Weinberg RS. [Diagnosis of intravascular lymphoma by a novel biopsy site.](http://www.ncbi.nlm.nih.gov/pubmed/21035865) *Ophthalmology.* 2011;118:586-590.
73. Lee BH, Pulido JS, Buettner H, Salomao D, Zent CS, Link TP. Intravascular B-cell lymphoma (angiotropic lymphoma) with choroidal involvement. *Arch Ophthalmol.* 2006;124:1357-1359.
74. Legeais M, Gallas S, Cottier JP, Herbreteau D. Paraplegia and sensory deficit caused by angiotropic large cell lymphoma. *AJNR Am J Neuroradiol*. 2004;25:1831-1835.
75. Levin KH, Lutz G. Angiotropic large-cell lymphoma with peripheral nerve and skeletal muscle involvement: early diagnosis and treatment. *Neurology.* 1996;47:1009-1011.
76. Liao JB, Hsieh PP, Hwang YC, Lin SL, Wu CS. [Cutaneous intravascular natural killer-cell lymphoma: a rare case and review of the literature.](http://www.ncbi.nlm.nih.gov/pubmed/21394417) *Acta Derm Venereol.* 2011;91:472-473.
77. Lie JT. Malignant angioendotheliomatosis (intravascular lymphomatosis) clinically simulating primary angiitis of the central nervous system. *Arthritis Rheum.* 1992;35:831-834.
78. Liew CL, Shyu WC, Tsao WL, Li H. [Intravascular lymphomatosis mimicks a cerebral demyelinating disorder.](http://www.ncbi.nlm.nih.gov/pubmed/17214091) *Acta Neurol Taiwan.* 2006;15:264-268.
79. Lim HW, Anderson HM. Angioendotheliomatosis associated with histiocytic lymphoma. Response to systemic chemotherapy. *J Am Acad Dermatol.* 1985;13:903-908.
80. Liszka U, Drlicek M, Hitzenberger P, et al. [Intravascular lymphomatosis: a clinicopathological study of three cases.](http://www.ncbi.nlm.nih.gov/pubmed/8263013) *J Cancer Res Clin Oncol*. 1994;120:164-168.
81. Liu H, Koyanagi I, Chiba H, et al. Spinal cord infarct as the initial clinical presentation of intravascular malignant lymphomatosis. *J Clin Neurosci.* 2009 ;16:570-573.
82. López-Gil F, Roura M, Umbert I, Umbert P. Malignant proliferative angioendotheliomatosis or angiotropic lymphoma associated with a soft-tissue lymphoma. *J Am Acad Dermatol*. 1992;26:101-104.
83. Lozsadi DA, Wieshmann U, Enevoldson TP. Neurological presentation of intravascular lymphoma: report of two cases and discussion of diagnostic challenges. *Eur J Neurol.* 2005;12:710-714.
84. Lu PH, Kuo TT, Yu KH, Lin TL, Chang SL, Yang CH. Intravascular large B-cell lymphoma presenting in subcutaneous fat tissue and simulating panniculitis clinically. *Int J Dermatol.* 2009 ;48:1349-1352.
85. Lui PC, Wong GK, Poon WS, Tse GM. Intravascular lymphomatosis. *J Clin Pathol.* 2003;56:468-470.
86. Ma X, Liu H. Intravascular large B-cell lymphoma originating in the left epididymis. *Ann Hematol*. 2011;90:107-108.
87. Madara J, Shane J, Scarlato M. Systemic endotheliomatosis: a case report. *J Clin Pathol.* 1975;28:476-482.
88. Maejima H, Tanei R, Morioka T, Miyakoshi S. Haemophagocytosis-related intravascular large B-cell lymphoma associated with skin eruption. *Acta Derm Venereol.* 2011;91:339-340.
89. Maisey NR, Waters JS, Collins D, Schofield J, Hill ME. A rare case of intravascular lymphoma diagnosed on bone marrow trephine. *Leuk Lymphoma.* 2003;44:1997-2000.
90. Malicki DM, Suh YK, Fuller GN, Shin SS. Angiotropic (intravascular) large cell lymphoma of T-cell phenotype presenting as acute appendicitis in a patient with acquired immunodeficiency syndrome. *Arch Pathol Lab Med.* 1999;123:335-337.
91. Manckoundia P, Rigaud-Royer I, Berthier S, et al. Intravascular malignant lymphomatosis diagnosed on a muscular biopsy: a case report. *Eur J Intern Med.* 2004;15:190-192.
92. Mandal AK, Savvidou L, Slater RM, Cockett W, Wiggins J, Missouris CG. Angiotropic lymphoma: Associated chromosomal abnormalities. *Eur J Intern Med.* 2007;18:432-434.
93. Marini-Bettolo C, Lane R, Charles P, et al. Myopathy secondary to intravascular large B-cell lymphoma. *Neuromuscul Disord.* 2009;19:856-859.
94. Martin-Duverneuil N, Mokhtari K, Behin A, Lafitte F, Hoang-Xuan K, Chiras J. [Intravascular malignant lymphomatosis.](http://www.ncbi.nlm.nih.gov/pubmed/12221446) *Neuroradiology.* 2002;44:749-754.
95. Martusewicz-Boros M, Wiatr E, Radzikowska E, Roszkowski-Sliz K, Langfort R. Pulmonary intravascular large B-cell lymphoma as a cause of severe hypoxemia. *J Clin Oncol*. 2007;25:2137-2139.
96. Masaki Y, Dong L, Nakajima A, et al. Intravascular large B cell lymphoma: proposed of the strategy for early diagnosis and treatment of patients with rapid deteriorating condition. *Int J Hematol*. 2009;89:600-610.
97. Massimino M, Giardini R, Cefalo G, et al. Intravascular lymphomatosis (IL) in a child mimicking a posterior fossa tumor. *J Neurooncol.* 2001;51:47-50.
98. Matsue K, Asada N, Odawara J, et al. Random skin biopsy and bone marrow biopsy for diagnosis of intravascular large B cell lymphoma. *Ann Hematol*. 2011;90:417-421.
99. Matsue K, Asada N, Takeuchi M, et al. A clinicopathological study of 13 cases of intravascular lymphoma: experience in a single institution over a 9-yr period. *Eur J Haematol.* 2008;80:236-244.
100. Matsue K, Takeuchi M, Uryu H, Koseki M, Asada N, Kaneko Y. Rapid improvement of hypoxemia by the use of rituximab in patients with pulmonary intravascular lymphoma. *Leuk Lymphoma.* 2007;48:197-200.
101. Merchant SH, Viswanatha DS, Zumwalt RE, Foucar K. Epstein-Barr virus-associated intravascular large T-cell lymphoma presenting as acute renal failure in a patient with acquired immune deficiency syndrome. *Hum Pathol.* 2003;34:950-954.
102. Mirza A, Torretti D, Tyler W, Pachipala K. Angiotropic large cell lymphoma presenting as fever of unknown origin. *Am J Hematol.* 2002;71:234-235.
103. Miura Y, Matsui Y, Sugino N, et al. Intravascular large B-cell lymphoma cells in the bone marrow smear preparation. *Br J Haematol.* 2011;152:237-238.
104. Miyazaki C, Mukai M, Kawaai Y, et al. A case of intravascular lymphoma with increased regional cerebral blood flow in I-123 IMP single-photon emission CT. AJNR *Am J Neuroradiol.* 2004;25:565-570.
105. Miyoshi I, Kubota T, Saito T, Toi M, Taguchi H. Intravascular lymphoma presenting with diverse neurologic manifestations. *Intern Med.* 2006;45:119-120.
106. Mleczko A, Franke I, Scheinpflug K, Gollnick H, Leverkus M. Intravascular large B-cell lymphoma: successful therapy with bendamustine and rituximab. *Acta Derm Venereol.* 2009;89:425-427.
107. [Mock DJ](http://www.ncbi.nlm.nih.gov/pubmed?term=Mock%20DJ%5BAuthor%5D&cauthor=true&cauthor_uid=8272931), [Jundt JW](http://www.ncbi.nlm.nih.gov/pubmed?term=Jundt%20JW%5BAuthor%5D&cauthor=true&cauthor_uid=8272931), [Green JB](http://www.ncbi.nlm.nih.gov/pubmed?term=Green%20JB%5BAuthor%5D&cauthor=true&cauthor_uid=8272931), Speights VO. Angiotropic lymphoma manifested by fever and painful swollen legs. *South Med J.* 1993;86:1432-1435.
108. Molina A, Lombard C, Donlon T. Immunohistochemical and cytogenetic studies indicate that malignant angioendotheliomatosis is a primary intravascular (angiotropic) lymphoma. *Cancer.* 1990;66:474-479.
109. Monteiro M, Duarte I, Cabecadas J, Orvalho ML. Intravascular large B-cell lymphoma of the breast. *Breast.* 2005;14:75-78.
110. Morikawa Y, Tohya K, Kuribayashi K , Saito K. A case of neoplastic angioendotheliomatosis: angiotropic lymphoma. *Appl Pathol.* 1989;7:322-328.
111. Morimoto K, Ogihara T, Shiomi T, Awaya N. Intravascular large B-cell lymphoma with preceding syndrome of inappropriate secretion of antidiuretic hormone. *Intern Med*. 2007;46:1569-1572.
112. Motegi S, Tamura A, Takeuchi Y, Ishikawa O. Senile angioma-like eruption: a skin manifestation of intravascular large B cell lymphoma. *Dermatology.* 2004;209:135-137.
113. Moussouttas M. Intravascular lymphomatosis presenting as posterior leukoencephalopathy. *Arch Neurol.* 2002;59:640-641.
114. Mudhar HS, Sethuraman C, Khan MD, Jan Su. Intraocular, pan-uveal intravascular large B-cell lymphoma associated with choroidal infarction and choroidal tri-lineage extramedullary haemtopoiesis. *Histopathology.* 2007;51:275-279.
115. Muftah S, Xu Z, El Gaddafi W, et al: [Synchronous intravascular large B-cell lymphoma within meningioma.](http://www.ncbi.nlm.nih.gov/pubmed/21615515) *Neuropathology.* Available from URL: http://onlinelibrary.wiley.com/doi/10.1111/j.1440-1789.2011.01223.x/full.
116. Murase T, Nakamura S, Kawauchi K, et al. An Asian variant of intravascular large B-cell lymphoma: clinical, pathological and cytogenetic approaches to diffuse large B-cell lymphoma associated with haemophagocytic syndrome. *Br J Haematol.* 2000;111:826-834.
117. Murase T, Nakamura S, Tashiro K, et al. Malignant histiocytosis-like B-cell lymphoma, a distinct pathologic variant of intravascular lymphomatosis: a report of five cases and review of the literature. *Br J Haematol.* 1997;99:656-664.
118. Murase T, Yamaguchi M, Suzuki R, et al. Intravascular large B-cell lymphoma (IVLBCL): a clinicopathologic study of 96 cases with special reference to the immunophenotypic heterogeneity of CD5. *Blood.* 2007;109:478-485.
119. Muto G, Takahashi Y, Yamashita H, Mimori A. A patient with intravascular lymphoma presenting with cerebral infarction and a high serum MPO-ANCA level. *Mod Rheumatol.* 2011;21:207-210.
120. Nagayama M, Shinohara Y, Sekiyama S, et al. Intravascular malignant lymphomatosis manifesting clinically as bilateral sudden hearing loss and cytomegalovirus encephalitis. *Neurology.* 1994;44:1518-1520.
121. Nakahara T, Saito T, Muroi A, et al. Intravascular lymphomatosis presenting as an ascending cauda equina: conus medullaris syndrome: remission after biweekly CHOP therapy. *J Neurol Neurosurg Psychiatry.* 1999;67:403-406.
122. Nakajima S, Ohshima K, Kyogoku M, Miyachi Y, Kabashima K. A case of intravascular large B-cell lymphoma with atypical clinical manifestations and analysis of CXCL12 and CXCR4 expression. *Arch Dermatol.* 2010;146:686-687.
123. Nakamichi N, Fukuhara S, Aozasa K, Morii E. NK-cell intravascular lymphomatosis--a mini-review. *Eur J Haematol*. 2008;81:1-7.
124. Nakamichi I, Shimazu K, Ikeda J, et al. Intravascular lymphomatosis initially suspected from uterine cytology: a case report. *Acta Cytol.* 2009;53:198-200.
125. Nakamura Y, Nakamagoe K, Kawachi Y, et al. Intravascular large B cell lymphoma with neurological symptoms diagnosed on the basis of a senile angioma-like eruption. *BMJ Case Rep.* Available from URL: http://www.ncbi.nlm.nih.gov/pmc/articles/PMC3029526/.
126. Nakamura T, Watanabe M, Hotchi M, Fujimori N, Mizuno M. Neoplastic angioendotheliomatosis. Report of two autopsy cases with special reference to the origin of atypical cells. *Acta Pathol Jpn*. 1987;37:1337-1346.
127. Nakanuma Y, Kumabashiri I. Neoplastic angioendotheliomatosis with multifocal hemorrhagic necrosis of the liver. *Am J Gastroenterol.* 1988;83:1180-1182.
128. Nakashima MO, Roy DB, Nagamine M, et al. Intravascular large B-cell lymphoma: a mimicker of many maladies and a difficult and often delayed diagnosis. *J Clin Oncol*. 2011;29:e138-140.
129. Narimatsu H, Morishita Y, Saito S, et al. [Usefulness of bone marrow aspiration for definite diagnosis of Asian variant of intravascular lymphoma: four autopsied cases.](http://www.ncbi.nlm.nih.gov/pubmed/15370213) *Leuk Lymphoma*. 2004;45:1611-1616.
130. Natali-Sora MG, Lodi M, Corbo M, Hays AP, Nemni R. Intravascular malignant lymphomatosis with neurological symptoms [letter]. *J Neurol.* 1996;243:205-206.
131. Niida T, Isoda K, Miyazaki K, et al. Pulmonary intravascular lymphoma diagnosed by 18-fluorodeoxyglucose positron emission tomography-guided transbronchial lung biopsy in a man with long-term survival: a case report. *J Med Case Reports.* 2011;7:295.
132. Niitsu N, Okamura D, Takahashi N, et al. Renal intravascular large B-cell lymphoma with early diagnosis by renal biopsy: a case report and review of the literature. *Leuk Res.* 2009;33:728-730.
133. Nishikawa K, Sekiyama S, Suzuki T, et al. A case of angiotropic large cell lymphoma manifesting nephrotic syndrome and treated successfully with combination chemotherapy. *Nephron.* 1991;58:479-482.
134. Nixon BK, Kussick SJ, Carlon MJ, Rubin BP. Intravascular large B-cell lymphoma involving hemangiomas: an unusual presentation of a rare neoplasm. *Mod Pathol.* 2005;18:1121-1126.
135. Odawara J, Asada N, Aoki T, et al. 18F-Fluorodeoxyglucose positron emission tomography for evaluation of intravascular large B-cell lymphoma. *Br J Haematol.* 2007;136:684.
136. Oei ME, Kraft GH, Sarnat HB. Intravascular lymphomatosis. *Muscle Nerve.* 2002;25:742-746.
137. Ohashi N, Aomatsu M, Mori A, et al. Intravascular lymphoma with extremely low high-density lipoproteinemia. *Intern Med*. 46:1475-1477.
138. Ohno T, Sakamoto T, Mizumoto C, et al. Leukemic and meningeal relapse of CD5+ intravascular large B-cell lymphoma with down-modulation of CD20 after rituximab therapy. *Int J Hematol*. 2006;84:74-78.
139. Okada Y, Nakanishi I, Nomura H, Takeda R, Nonomura A, Takekuma K. Angiotropic B-cell lymphoma with hemophagocytic syndrome. *Pathol Res Pract.* 1994;190:718-724.
140. Okagaki T, Richart RM. Systemic proliferating angioendotheliomatosis. A case report. *Obstet Gynecol.* 1971;37:377-380.
141. Ormsby A, Prayson R, Heard R. Angiotropic large cell lymphoma mimicking multiple sclerosis associated transverse myelitis. *J Clin Neurosci.* 1999;6:408-410.
142. Ossege LM, Postler E, Pleger B, Muller KM, Malin JP. Neoplastic cells in the cerebrospinal fluid in intravascular lymphomatosis. *J Neurol.* 2000;247:656-658.
143. Otrakji CL, Voigt W, Amador A, Nadjil M, Gregorios JB. Malignant angioendotheliomatosis--a true lymphoma: a case of intravascular malignant lymphomatosis studied by southern blot hybridization analysis. *Hum Pathol.* 1988;19:475-478.
144. Owa M, Koyama J, Asakawa K, Hikita H, Lubo K, Ikeda SI. Intravascular lymphomatosis presenting as reversible severe pulmonary hypertension. *Int J Cardiol.* 2000;75:283-284.
145. Ozguroglu E, Buyulbabani N, Ozguroglu M, Baykal C. Generalized telangiectasia as the major manifestation of angiotropic (intravascular) lymphoma. *Br J Dermatol.* 1997;137:422-425.
146. Pahk PJ, Todd DJ, Blaha GR, et al. Intravascular lymphoma masquerading as Vogt-Koyanagi-Harada syndrome. *Ocul Immunol Inflamm.* 2008;16:123-126.
147. Papalas JA, Proia AD, Cummings TJ. Hematolymphoid malignancies with intraocular intravascular involvement: report of 2 cases. *Ann Diagn Pathol.* 2011;15:286-290.
148. Park GH, Kim CH, Chung WK, et al. Primary cutaneous intravascular large B-cell lymphoma treated with combination chemotherapy and complicated by rituximab-induced interstitial lung disease*. Acta Derm Venereol.* 2010;90:296-298.
149. Park JH, Lee DY, Ko YH. [Intravascular large B-cell lymphoma of the cutaneous variant in Korea.](http://www.ncbi.nlm.nih.gov/pubmed/21269312) *J Dermatol.* 2011;38:160-163.
150. Parrens M, Dubus P, Agape P, et al: Intrasinusoidal bone marrow infiltration revealing intravascular lymphomatosis. *Leuk Lymphoma.* 37:219-23, 2000
151. Passarin MG, Wen PY, Vattemi E, et al. [Intravascular lymphomatosis and intracerebral haemorrhage.](http://www.ncbi.nlm.nih.gov/pubmed/20517703) *Neurol Sci.* 2010;31:793-797.
152. Pasyk K, Depowski M. Proliferating systematized angioendotheliomatosis of a 5 month old infant. *Arch Dermatol.* 1978;114:1512-1515.
153. Pekic S, Milicevic S, Colovic N, Colovic M, Popovic V. Intravascular large B-cell lymphoma as a cause of hypopituitarism: gradual and late reversal of hypopituitarism after long-term remission of lymphoma with immunochemotherapy. *Endocrine.* 2008;34:11-16.
154. Pellicone JT, Goldstein HB. [Pulmonary malignant angioendotheliomatosis. Presentation with fever and syndrome of inappropriate antidiuretic hormone.](http://www.ncbi.nlm.nih.gov/pubmed/2225987) *Chest.* 1990;98:1292-1294.
155. Perniciaro C, Winkelmann RK, Daoud MS, Su WP. Malignant angioendotheliomatosis is an angiotropic intravascular lymphoma. Immunohistochemical, ultrastructural, and molecular genetics studies. Am J Dermatopathol 1995;17:242-248.
156. Petroff N, Koger OW, Fleming MG, et al. Malignant angioendotheliomatosis: an angiotropic lymphoma. *J Am Acad Dermatol.* 1989;21:727-733.
157. Piyatanont K, Bamrungrak K, Watcharananan S, et al. Intravascular B-cell lymphoma presenting with cauda equina syndrome: the role of skin biopsy. *Eur J Dermatol.* 2010;20:821-822.
158. Ponzoni M, Arrigoni G, Gould VE, et al. Lack of CD 29 (beta1 integrin) and CD 54 (ICAM-1) adhesion molecules in intravascular lymphomatosis. *Hum Pathol.* 2000;31:220-226.
159. Prayson RA. [Angiotropic large cell lymphoma: simultaneous peripheral nerve and skeletal muscle involvement.](http://www.ncbi.nlm.nih.gov/pubmed/8714265) *Pathology.* 1996;281:25-27.
160. Prayson RA, Segal GH, Stoler MH, Licata AA, Tubbs RR. Angiotropic large-cell lymphoma in a patient with adrenal insufficiency. *Arch Pathol Lab Med.* 1991;115:1039-1041.
161. Price DA, Thaker H, James A, Snow MH. Hypopituitarism in a patient with intravascular lymphomatosis. *Haematologica.* 2002;87:ECR36.
162. Pusch G, Feher G, Szomor A, Kover F, Gomori E, Illes Z. Intravascular lymphoma presenting with neurological signs but diagnosed by prostate biopsy: suspicion as a key to early diagnosis. *Eur J Neurol.* 2009;16:e39-41.
163. Quadri AM, Sylvester S, Verma S, et al. Angiotropic large cell lymphoma presenting as intestinal obstruction. *Ann Hematol.* 2008;87:67-69.
164. Rahman F, Pittaluga S. [Intravascular lymphoma.](http://www.ncbi.nlm.nih.gov/pubmed/19670496) *Blood.* 2009;114:1140.
165. Raroque HG Jr, Mandler RN, Griffey MS, Orrison WW, Kornfield M. [Neoplastic angioendotheliomatosis.](http://www.ncbi.nlm.nih.gov/pubmed/2375700) *Arch Neurol.* 1990;47:929-930.
166. Rashid R, Johnson RJ, Morris S, et al. Intravascular large B-cell lymphoma associated with a near-tetraploid karyotype, rearrangement of BCL6, and a t(11;14)(q13;q32). *Cancer Genet Cytogenet.* 2006;171:101-104.
167. Remberger K, Nawrath-Koll I, Gokel JM, Haider M. [Systemic angioendotheliomatosis of the lung.](http://www.ncbi.nlm.nih.gov/pubmed/3299327) *Pathol Res Pract.* 1987;182:265-274.
168. Rieger KE, Polidore T, Warnke R, Kim J. ALK-negative systemic intravascular anaplastic large cell lymphoma presenting in the skin. *J Cutan Pathol.* 2011;38:216-220.
169. Rieger E, Soyer HP, Leboit PE, Metze D, Slovak R, Keri H. [Reactive angioendotheliomatosis or intravascular histiocytosis? An immunohistochemical and ultrastructural study in two cases of intravascular histiocytic cell proliferation.](http://www.ncbi.nlm.nih.gov/pubmed/10233274) *Br J Dermatol.* 1999;140:497-504.
170. Rose C, Staumont D, Jouet JP. Successful autologous bone marrow transplantation in intravascular lymphomatosis [letter]. *Br J Haematol.* 1999;105:313-314.
171. Roshal M, Till BG, Fromm JR, Cherian S. Intravascular large B cell lymphoma presenting in a liver explant. *J Clin Pathol.* 2008;61:877-878.
172. Roussou P, Ilias I, Fotinou M. Angiotropic lymphoma: a rare hematological malignancy. *Haematologica.* 1998;83:376.
173. Roux S, Grossin M, De Bandt M, Palazzo E, Vachon F, Kahn MF. Angiotropic large cell lymphoma with mononeuritis multiplex mimicking systemic vasculitis. *J Neurol Neurosurg Psychiatry*. 1995;58:363-366.
174. Rubin MA, Cossman J, Freter CE, Azumi N. Intravascular large cell lymphoma coexisting within hemangiomas of the skin. *Am J Surg Pathol.* 1997;21:860-864.
175. Rubio A, Poole RM, Brara HS, Taylor N, Powers JM. Motor neuron disease and angiotropic lymphoma. *Arch Neurol;* 1997;54:92-95.
176. Sajid RM, Qureshi A. Involvement of bone marrow with intravascular large B-cell lymphoma. *Hematol Oncol Stem Cell Ther.* 2010;3:39-41.
177. Saleh Z, Kurban M, Ghosn S, Awar G, Kibbi AG. Generalized telangiectasia: a manifestation of intravascular B-cell lymphoma. *Dermatology.* 2008;217:318-320.
178. Sánchez-Cano D, Callejas-Rubio JL, Vilanova-Mateu A, Gomez-Morales M, Ortego-Centeno N. Intravascular lymphoma in a patient with systemic lupus erythematosus: a case report. *Lupus.* 2010;16:525-528.
179. Sangueza O, Hyder DM, Sangueza P. Intravascular lymphomatosis: report of an unusual case with T cell phenotype occurring in an adolescent male. *J Cutan Pathol.* 1992;19:226-231.
180. Sanna P, Bertoni F, Roggero E,et al. Angiotropic (intravascular) large cell lymphoma: case report and short discussion of the literature. *Tumori.* 1997;83:772-775.
181. Satoh S, Yamazaki M, Yahikozawa H, et al. Intravascular large B cell lymphoma diagnosed by senile angioma biopsy. *Intern Med*. 2003;42:117-120.
182. Satti S, Castillo R. Intravascular B-Cell Lymphoma. *Community Oncology.* 2005;2:55–60.
183. Saurel CA, Personett DA, Edenfield BH, et al. Molecular analysis of intravascular large B-cell lymphoma with neoangiogenesis. *Br J Haematol* 2011;152:234-236.
184. Savard M, Verreault S, Gould PV, Bernier V, Bouchard JP. Intravascular lymphoma with conus medullaris syndrome followed by encephalopathy. Can J Neurol Sci 2008;35:366-371.
185. Savarese DM, Zavarin M, Smyczynski MS, Rohrer MJ, Hutzier MJ. Super vena cava syndrome secondary to an angiotropic large cell lymphoma. *Cancer.* 2000;89:2515-2520.
186. Sawa N, Ubara Y, Katori H, et al. Renal intravascular large B-cell lymphoma localized only within peritubular capillaries. Report of a case. *Intern Med*. 2007;46:657-662.
187. Sawamoto A, Narimatsu H, Suzuki T, Kurahashi S, Sugimoto T, Sugiura I. Long-term remission after autologous peripheral blood stem cell transplantation for relapsed intravascular lymphoma. *Bone Marrow Transplant.* 2006;37:233-234.
188. Schonfeld SM, Pinto RS, Aleksic S, Pearson J. Cerebral angioendotheliomatosis: a report of two cases and review of the literature. *AJNR Am J Neuroradiol.* 1985;6:437-441.
189. Schleinitz N, Bernit E, Mazodier K et al. Two cases of intravascular lymphomatosis revealed by hypopituitarism. *Haematologica.* 2002;87:ECR21.
190. Schwartz S, Zoubaa S, Knauth M, Sommer C, Storch-Hagenlocher B. Intravascular lymphomatosis presenting with a conus medullaris syndrome mimicking disseminated encephalomyelitis. *Neuro Oncol*. 2002;4:187-191.
191. Scott PW, Silvers DN, Helwig EB. Proliferating angioendotheliomatosis. *Arch Pathol.* 1975;99:323-326.
192. [Seki K](http://www.ncbi.nlm.nih.gov/pubmed?term=Seki%20K%5BAuthor%5D&cauthor=true&cauthor_uid=15371956), [Miyakoshi S](http://www.ncbi.nlm.nih.gov/pubmed?term=Miyakoshi%20S%5BAuthor%5D&cauthor=true&cauthor_uid=15371956), [Lee GH](http://www.ncbi.nlm.nih.gov/pubmed?term=Lee%20GH%5BAuthor%5D&cauthor=true&cauthor_uid=15371956), et al. Prostatic acid phosphatase is a possible tumor marker for intravascular large B-cell lymphoma. *Am J Surg Pathol.* 2004;28:1384-1388.
193. Sengupta S, Pedersen NP, Davis JE, et al. Illusion of stroke intravascular lymphomatosis. *Rev Neurol Dis* 2011;8:e107-113.
194. Sepandj F, Gupta R, Foyle A. Renal manifestations of angiotrophic lymphoma clinicopathological features. *Nephrol Dial Transplant.* 1997;12:190-194.
195. Sepp N, Schuler G, Romani N, et al. "Intravascular lymphomatosis" (angioendotheliomatosis): evidence for a T-cell origin in two cases. *Hum Pathol.* 1990;21:1051-1058.
196. Setoyama M, Mizoguchi S, Orikawa T, Tashiro M. A case of intravascular malignant lymphomatosis (angiotropic large-cell lymphoma) presenting memory T cell phenotype and its expression of adhesion molecules. *J Dermatol.* 1992;19:263-269.
197. Shan SJ, Chen J, Geng SL, et al. Successful treatment of cutaneous intravascular large B-cell lymphoma with fludarabine phosphate. *Eur J Dermatol.* 2010;20:408-409.
198. [Sheibani K](http://www.ncbi.nlm.nih.gov/pubmed?term=Sheibani%20K%5BAuthor%5D&cauthor=true&cauthor_uid=3485768), [Battifora H](http://www.ncbi.nlm.nih.gov/pubmed?term=Battifora%20H%5BAuthor%5D&cauthor=true&cauthor_uid=3485768), [Winberg CD](http://www.ncbi.nlm.nih.gov/pubmed?term=Winberg%20CD%5BAuthor%5D&cauthor=true&cauthor_uid=3485768), et al. Further evidence that "malignant angioendotheliomatosis" is an angiotropic large-cell lymphoma. *N Eng J Med.* 1986;314:943-948.
199. Shen Q, Duan X, Feng W, et al. Intravascular large B-cell lymphoma: report of three cases and analysis of the mTOR pathway. *Int J Clin Exp Pathol.* 2011;4:782-790.
200. Shimada K, Kosugi H, Narimatsu H, et al. Sustained remission after rituximab-containing chemotherapy for intravascular large B-cell lymphoma. *J Clin Exp Hematop.* 2008;48:25-28.
201. Shimada K, Kosugi H, Shimada S, et al. Evaluation of organ involvement in intravascular large B-cell lymphoma by 18F-fluorodeoxyglucose positron emission tomography. *Int J Hematol.* 2008;88:149-153.
202. Shimada K, Murase T, Matsue K, et al. Central nervous system involvement in intravascular large B-cell lymphoma: a retrospective analysis of 109 patients. *Cancer Sci.* 2010;101:1480-1486.
203. Shimizu I, Ichikawa N, Yotsumoto M, Sumi M, Ueno M, Kobayashi H. Asian variant of intravascular lymphoma: aspects of diagnosis and the role of rituximab. *Intern Med.* 2007;46:1381-1386.
204. Shimokawa I, Higami Y, Sakai H, Moriuchi Y, Murase K, Ikeda T. Intravascular malignant lymphomatosis: a case of T-cell lymphoma probably associated with human T-cell lymphotropic virus. *Hum Pathol* 1991;22:200-202.
205. Shiraki K, Sugimoto K, Deguchi M, Ito N, Masuda C, Takei Y. Hepatic intravascular large B cell lymphoma. *Intern Med*. 2007;46:1761-1762.
206. Sill H, Hofler G, Kaufmann P, et al. Angiotropic large cell lymphoma repsenting thrombotic microangiopathy (thrombotic thrombocytopenia purura). *Cancer.* 1995;1:1167-1170.
207. Sips GJ, Amory CF, Delman BN, Kleinman GM, Lipsey LR, Tuhrim S. [Intravascular lymphomatosis of the brain in a patient with myelodysplastic syndrome.](http://www.ncbi.nlm.nih.gov/pubmed/19488086) *Nat Rev Neurol.* 2009;5:288-292.
208. Sleater JP, Segal GH, Scott MD, Masih AS. Intravascular (angiotropic) large cell lymphoma: determination of monoclonality by polymerase chain reaction on paraffin-embedded tissues. *Mod Pathol*. 1994;7:593-598.
209. Smadja D, Mas JL, Fallet-Bianco C, et al. Intravascular lymphomatosis (neoplastic angioendotheliosis) of the central nervous system: case report and literature review. *J Neurooncol.* 1991;11:171-180.
210. Smith ME, Stamatakos MD, Neuhauser TS. Intravascular lymphomatosis presenting within angiolipomas. *Ann Diagn Pathol.* 2001;5:103-106.
211. Snowden JA, Angel CA, Winfield DA, Pringle JH, West KP. [Angiotropic lymphoma: report of a case with histiocytic features.](http://www.ncbi.nlm.nih.gov/pubmed/9059361) *J Clin Pathol.* 1997;50:67-70.
212. Snyder LS, Harmon KR, Estensen RD. [Intravascular lymphomatosis (malignant angioendotheliomatosis) presenting as pulmonary hypertension.](http://www.ncbi.nlm.nih.gov/pubmed/2805852) *Chest.* 1989;96:1199-1200.
213. Sokol DK, Azzarelli B, Smith RR et al. Primary intravascular lymphomatosis associated with mycobacterium marinum. *J Neuroimag.* 1998;8:47-49.
214. Song DK, Boulis NM, McKeever PE, Quint DJ. Angiotropic large cell lymphoma with imaging characteristics of CNS vasculitis. *AJNR Am J Neuroradiol.* 2002;23:239-242.
215. Song DE, Lee MW, Ryu MH, Kang DW, Kim SJ, Huh J. Intravascular large cell lymphoma of the natural killer cell type. J Clin Oncol 2007;25:1279-1282.
216. Souza CA, Quan K, Seely J, Kravcik S, Burns B. Pulmonary intravascular lymphoma. *J Thorac Imaging.* 2009;24:231-233.
217. Srivatsa S, Sharma J, Logani S. Intravascular lymphoma: an unusual diagnostic outcome of an incidentally detected adrenal mass. *Endocr Pract.* 2008;14:884-888.
218. Stahl RL, Chan W, Duncan A, Corley CC Jr. [Malignant angioendotheliomatosis presenting as disseminated intravascular coagulopathy.](http://www.ncbi.nlm.nih.gov/pubmed/1913468) *Cancer.* 2008;68:2319-2323.
219. Stroup RM, Sheibani K, Moncada A, Purdy LJ, Battifora H. [Angiotropic (intravascular) large cell lymphoma. A clinicopathologic study of seven cases with unique clinical presentations.](http://www.ncbi.nlm.nih.gov/pubmed/1698530) *Cancer.* 1990;66:1781-1788.
220. Suarez-Vilela D, Izquierdo-Garcia FM, Ramos-Ortega F. Intravascular lymphomatosis in T-hepatosplenic lymphoma. *Am J Clin Pathol* 2002;117:662-663.
221. Sukpanichnant S, Visuthisakchai S. Intravascular lymphomatosis: study of 20 cases in Thailand and a review of the literature. *Clin Lymphoma Myeloma* 2006;6:319-328.
222. Sugimoto KJ, Mori KL, Oshimi K. Intravascular large B-cell lymphoma. *Am J Hematol.* 2004;76:291-292.
223. Suh CH, Kim SK, Shin DH, Chung KY, Kim SK. [Intravascular lymphomatosis of the T cell type presenting as interstitial lung disease--a case report.](http://www.ncbi.nlm.nih.gov/pubmed/9364306) *J Korean Med Sci.* 1997;12:457-460.
224. Sumer M, Ozon AO, Bakar B, Cila A, Ruacan S. Intravascular lymphoma masquerading as multiembolic stroke developing after coronary artery by-pass surgery. *The Neurologist.* 2009;15:98-101.
225. Sur M, Ross C, Moens F, Daya D. Intravascular large B-cell lymphoma of the uterus: a diagnostic challenge. *Int J Gynecol Pathol.* 2005;24:201-203.
226. Suzuki S, Koizumi Y. Angiotropic lymphoma diagnosed by muscle biopsy. *Intern Med.* 1997;36:304-307.
227. Suzumiya J, Ohshima K, Kanda M, et al. Intravascular large cell lymphoma associated with hypoalbuminemia. *Leuk Lymphoma.* 1998;32:179-182.
228. Svajdler M, Lazúrová I, Bohus P, Pal’ko M. Intravascular variant of diffuse large B-cell lymphoma with combined endocrine involvement. *Wien Klin Wochenschr*. 2006;118:422-425.
229. Szots M, Szomor A, Kover F, et al. Intravascular lymphomatosis of the nervous system. *J Neurol.* 2008;255:1590-1592.
230. Szuba A, Koba M, Rzeszutko M, et al. Cutaneous angiogenesis in patient with intravascular lymphoma (IVL): A case report. *Dermatol Online J.* 2010;16:2.
231. Takacs I, Eros N, Bene I, et al. Successful treatment of relapse of an intravascular B-cell lymphoma with rituximab-CHOP polychemotherapy. *Ann Hematol.* 2004;83:608-610.
232. Takahashi E, Kajimoto K, Fukatsu T, Yoshida M, Elmoto T, Nakamura S. Intravascular large T-cell lymphoma: a case report of CD30-positive and ALK-negative anaplastic type with cytotoxic molecule expression. *Virchows Arch.* 2005;447:1000-1006.
233. Takahashi T, Minato M, Tsukuda H, Yoshimoto M, Tsujisaki M. Successful treatment of intravascular large B-cell lymphoma diagnosed by bone marrow biopsy and FDG-PET scan. *Intern Med*. 2008;47:975-979.
234. Takamura K, Nasuhara Y, Mishina T, et al. Intravascular lymphomatosis diagnosed by transbronchial lung biopsy. *Eur Respir J.* 1997;10:955-957.
235. Takizawa S, Shirasugi Y, Nakamura N, et al. An atypical form of Asian variant of intravascular large B-cell lymphoma presenting with myelopathy alone for 4 months prior to pancytopenia. *Intern Med*. 2007;46:1879-1880.
236. Tan TB, Spaander PJ, Blaisse M, Gerritzen Fm. Angiotropic large cell lymphoma presenting as interstitial lung disease. *Thorax.* 1988;43:578-579.
237. Terrier B, Aouba A, Vasiliu V, et al. Intravascular lymphoma associated with haemophagocytic syndrome: a very rare entity in western countries. *Eur J Haematol.* 2005;75:341-345.
238. Thomson JJ, Walt JV, Ireland R. Bone marrow trephine biopsy appearances of the intravascular subtype of diffuse large B-cell lymphoma. *Br J Haematol.* 2007;136:683.
239. Tokura T, Murase T, Toriyama T, et al. Asian variant of CD5+ intravascular large B-cell lymphoma with splenic infarction. *Intern Med*. 2003;42:105-109.
240. Tomasini C, Novelli M, Ponti R, Pippione M, Bernengo MG. Cutaneous intravascular lymphoma following extravascular lymphoma of the lung. *Dermatology.* 2004;208:158-163.
241. Torenbeek R, Scheltens P, Strack van Schijindel RJ, Algra PR, Heimans JJ, van der Valk P. Angiotropic intravascular large-cell lymphoma with massive cerebral extension. *J Neurol Neurosurg Psychiatry.* 1993;56:914-916.
242. Tranchida P, Bayerl M, Voelpel MJ, Palutke M. Testicular ischemia due to intravascular large B-cell lymphoma: a novel presentation in an immunosuppressed individual. *Int J Surg Pathol.* 2003;11:319-324.
243. Treves TA, Gadoth N, Blumen S, Korczyn AD. Intravascular malignant lymphomatosis: a cause of subacute dementia. *Dementia.* 1995;6:286-293.
244. [Tsukadaira A](http://www.ncbi.nlm.nih.gov/pubmed?term=Tsukadaira%20A%5BAuthor%5D&cauthor=true&cauthor_uid=11943898), [Okubo Y](http://www.ncbi.nlm.nih.gov/pubmed?term=Okubo%20Y%5BAuthor%5D&cauthor=true&cauthor_uid=11943898), [Ogasawara H](http://www.ncbi.nlm.nih.gov/pubmed?term=Ogasawara%20H%5BAuthor%5D&cauthor=true&cauthor_uid=11943898), et al. Chromosomal aberrations in intravascular lymphomatosis. *Am J Clin Oncol.* 2002;25:178-181.
245. Tucker TJ, Bardales RH, Miranda RN. Intravascular lymphomatosis with bone marrow involvement. *Arch Pathol Lab Med.* 1999;123:952-956.
246. Van Droogenbroeck J, Altintas S, Pollefliet C, Schroyens W, Bernerman Z. Intravascular large B-cell lymphoma or intravascular lymphomatosis: report of a case diagnosed by testicle biopsy. *Ann Hematol.* 2001;80:316-318.
247. Vandenheede M, Dioh A, de Noordhout AM, Deprez M, Schoenen J. Intravascular malignant lymphomatosis: report of 2 neurological cases. *Acta Neurol Belg.* 2002;102:76-81.
248. Venizelos I, Tamiolakis D, Petrakis G. High grade primary adrenal intravascular large B-cell lymphoma manifesting as Addison disease. *Rev Esp Enferm Dig.* 2007;99:471-474.
249. Viali S, Hutchinson DO, Hawkins TE, et al. Presentation of intravascular lymphomatosis as lumbosacral polyradiculopathy. *Muscle Nerve.* 2000;23:1295-1300.
250. Vital C, Vital A, Julien J, et al. Peripheral neuropathies and lymphoma without monoclonal gammopathy: a new classification. *J Neurol.* 1990;237:177-185.
251. Vieites B, Fraga M, Lopez-Presas E, Pintoz E, Garcia-Rivero A, Forteza J. Detection of t(14;18) translocation in a case of intravascular large B-cell lymphoma: a germinal centre cell origin in a subset of these lymphomas? *Histopathology.* 2005;46:466-468.
252. Vieren M, Sciot R, Robberecht W. Intravascular lymphomatosis of the brain: a diagnostic problem. *Clin Neurol Neurosurg.* 1999;101:33-36.
253. Von Kempis J, Kohler G, Herbst EW, Peter HH. Intravascular lymphoma presenting as symmetric polyarthritis. *Arthritis Rheum.* 1998;41:1126-1130.
254. Vos JM, Bordbar A, Vet RJ, Pals ST, Kater AP. Waxing and waning intravascular large cell lymphoma with widespread organ infiltration. *Leuk Lymphoma.* 2011;52:705-708.
255. Vougiouklakis T, Mitselou A, Agnantis NJ. Angiotropic large cell lymphoma. *J Exp Clin Cancer Res.* 2004;23:345-348.
256. Vrindavanam N, Hamadani M, Steele B, Awan F, Suster S, Benson DM Jr. Dramatic response to single-agent rituximab in a patient with intravascular lymphoma. *Am J Hematol*. 2007;82:1120-1121.
257. Wach M, Dmoszynska A, Skomra D, Wasik-Szczepanek E, Szumilo J. Intravascular B-cell lymphoma in a 38-year-old woman: a case report. *Ann Hematol.* 2001;80:224-227.
258. Wahie S, Dayala S, Husain A, et al. Cutaneous features of intravascular lymphoma. *Clin Exp Dermatol.* 2011;36:288-291.
259. Wake A, Kakinuma A, Mori N, et al. Angiotropic lymphoma of paranasal sinuses with initial symptoms of oculomotor nerve palsy*. Intern Med.* 1993;32:237-242.
260. Walker UA, Herbst EW, Ansorge O, Peter HH. Intravascular lymphoma simulating vasculitis *Rheumatol Int.* 1994;14:131-133.
261. Walls JG, Hong YG, Cox JE, et al. [Pulmonary intravascular lymphomatosis: presentation with dyspnea and air trapping.](http://www.ncbi.nlm.nih.gov/pubmed/10208234) *Chest.* 1999;115:1207-1210.
262. Wang L, Li C, Gao T. Cutaneous intravascular anaplastic large cell lymphoma. *J Cutan Pathol.* 2011;38:221-226.
263. Wang BY, Strauchen JA, Rabinowitz D, Tillem SM, Unger PD. Renal cell carcinoma with intravascular lymphomatosis: a case report of unusual collision tumors with review of the literature. *Arch Pathol Lab Med* 2001;125:1239-1241.
264. Waring WS, Wharton SB, Grant R, McIntyre M. Angiotropic large B-cell lymphoma with clinical features resembling subacute combined degeneration of the cord. *Clin Neurol Neurosurg.* 1999;101:275-279.
265. [Watabe R](http://www.ncbi.nlm.nih.gov/pubmed?term=Watabe%20R%5BAuthor%5D&cauthor=true&cauthor_uid=11100751), [Shibata K](http://www.ncbi.nlm.nih.gov/pubmed?term=Shibata%20K%5BAuthor%5D&cauthor=true&cauthor_uid=11100751), [Hirase N](http://www.ncbi.nlm.nih.gov/pubmed?term=Hirase%20N%5BAuthor%5D&cauthor=true&cauthor_uid=11100751), et al. Angiotropic B-cell lymphoma with hemophagocytic syndrome associated with syndrome of inappropriate secretion of antidiuretic hormone. *Ann Hematol.* 2000;79:581-584.
266. Weichert G, Martinka M, Rivers JK. Intravascular lymphoma presenting as telangectasias: response to rituximab and combination chemotherapy. *J Cutan Med Surg*. 2003;7:460-463.
267. Weisel KC, Brugger W, Krober SM, Kaiserling E, Kanz L. Intravascular lymphoma - a rare cause of hemolytic anemia and neurologic disorders. *Hematol J*. 2004;5:444-446.
268. Weitten T, Guiot P, Mootien Y, Rozan-Rodier S, Andres E. Multiorgan failure caused by intravascular lymphoma. *QJM.* 2008;101:508-510.
269. [Wick MR](http://www.ncbi.nlm.nih.gov/pubmed?term=Wick%20MR%5BAuthor%5D&cauthor=true&cauthor_uid=7296500), [Banks PM](http://www.ncbi.nlm.nih.gov/pubmed?term=Banks%20PM%5BAuthor%5D&cauthor=true&cauthor_uid=7296500), [McDonald TJ](http://www.ncbi.nlm.nih.gov/pubmed?term=McDonald%20TJ%5BAuthor%5D&cauthor=true&cauthor_uid=7296500). Angioendotheliomatosis of the nose with fatal systemic dissemination. *Cancer.* 1981;48:2510-2517.
270. Wick MR, Scheithauer BW, Okazaki H, Thomas JE. Cerebral angioendotheliomatosis. *Arch Pathol Lab Med.* 1982;106:342-346.
271. Willemze R, Kruyswijk MR, De Bruin CD, Meijer CJ, Van Berkel W. [Angiotropic (intravascular) large cell lymphoma of the skin previously classified as malignant angioendotheliomatosis.](http://www.ncbi.nlm.nih.gov/pubmed/2952157) *Br J Dermatol.* 1987;116:393-399.
272. Williams G, Foyle A, White D, Greer W, Burrell S, Couban S. Intravascular T-cell lymphoma with bowel involvement: case report and literature review. *Am J Hematol.* 2005;78:207-211.
273. Williams DB, Lyons MK, Yanagihara T, Coglan JP, Banks PM. Cerebral angiotropic large cell lymphoma (neoplastic angioendotheliosis): therapeutic considerations. *J Neurol Sci.* 1991;103:16-21.
274. Williams RE, Seywright MM, Lever R, Lucie NP. Angiotropic B-cell lymphoma (malignant angioendotheliomatosis): failure of systemic chemotherapy. *Br J Dermatol*. 1990;123:807-810.
275. Wong P, Moonie A, Dennett X, Anpalahan M. A case of intravascular lymphomatosis (IVL) presenting as polyneuropathy. *Eur J Intern Med*. 2006;17:59-60.
276. Wrotnowski U, Mills SE, Cooper PH. Malignant angioendotheliomatosis. An angiotropic lymphoma? *Am J Clin Pathol.* 1985;83:244-248.
277. Wu SJ, Chou WC, Ko BS, Tien HF. Severe pulmonary complications after initial treatment with Rituximab for the Asian-variant of intravascular lymphoma. *Haematologica.* 2007;92:141-142.
278. Wu H, Said JW, Ames ED, et al. First reported cases of intravascular large cell lymphoma of the NK cell type: clinical, histologic, immunophenotypic, and molecular features. *Am J Clin Pathol.* 2005;123:603-611.
279. Xanthopoulos V, Galanopoulos AG, Paterakis G, et al. Intravascular B-cell lymphoma with leukemic presentation: case reportand literature review. *Eur J Haematol*. 2008;80:177-181.
280. Xia C, Lang SY, Li XM, Yu SY. Intravascular lymphomatosis with recurrent cerebral hemorrhages. *Neurol India.* 2009;57:817-819.
281. Xu M, Yang Q, Li M, Geng W, Huang W, Chen Y. Prostate involvement by intravascular large B-cell lymphoma: a case report with literature review. *Int J Surg Pathol.* 2011;19:544-547.
282. Yalamanchili M, Prabhu S, Bradstreet P, Chouhdry I, Walsh J. Intravascular lymphomatosis presenting as systemic inflammatory response syndrome. *Am J Med Sci.* 2002;324:339-341.
283. Yamada S, Nishii R, Oka S, et al. FDG-PET a pivotal imaging modality for diagnosis of stroke-onset intravascular lymphoma. *Arch Neurol.* 2010;67:366-367.
284. Yamada N, Uchida R, Fuchida S, et al. CD5+ Epstein-Barr virus-positive intravascular large B-cell lymphoma in the uterus co-existing with huge myoma. *Am J Hematol.* 2005;78:221-224.
285. Yamagata T, Okamoto Y, Ota K, Katayama N, Tsuda T, Yukawa S. A case of pulmonary intravascular lymphomatosis diagnosed by thoracoscopic lung biopsy. *Respiration.* 2003;70:414-418.
286. Yamaguchi M, Kimura M, Watanabe Y, et al. Successful autologous peripheral blood stem cell transplantation for relapsed intravascular lymphomatosis. *Bone Marrow Transplant.* 2001;27:89-91.
287. Yamamoto T, Morita K, Iriyama N, et al. Intravascular large B-cell lymphoma of the uterus: a case with favorable clinical outcome. *Int J Surg Pathol.* 2011;19:672-676.
288. Yanagihori H, Oyama N, Kawakami Y, et al. A case of intravascular large B-cell lymphoma with multiple organ involvement. *J Dermatol.* 2003;30:910-914.
289. Yang T, Tian L, Li Q, et al. A case of intravascular B-cell lymphoma presenting as myelopathy and diagnosed post mortem. *J Neurol Sci.* 2008;272:196-198.
290. Yao X, Saad A, Chitambar CR. [Intravascular large B-cell lymphoma, an exclusively small vessel disease? A case report and review of literature.](http://www.ncbi.nlm.nih.gov/pubmed/20542564) *Leuk Res.* 2010;34:e275-277.
291. Yasuda M, Akiyama N, Miyamoto S, et al. Primary sellar lymphoma: intravascular large B-cell lymphoma diagnosed as a double cancer and improved with chemotherapy, and literature review of primary parasellar lymphoma. *Pituitary.* 2010;13:39-47.
292. Yasuda H, Ando J, Matsumoto T, et al. [Intravascular large B cell lymphoma with hepatic portal vein, splenic vein and mesenteric vein tumour embolism.](http://www.ncbi.nlm.nih.gov/pubmed/20955393) *Histopathology.* 2010;57:648-650.
293. Yegappan S, Coupland R, Arber DA, et al. Angiotropic lymphoma: an immunophenotypically and clinically heterogeneous lymphoma. *Mod Pathol*. 2001;14:1147-1156.
294. Yeung CK, Trendell-Smith NL, Mak HK, et al. 'Western' or 'Asian' intravascular large B-cell lymphoma? *Clin Exp Dermatol.* 2009;34:e482-483.
295. Yin W, Li M, Gao Z, Huang F, Da J, Liu C. Intravascular large B-cell lymphoma with involvement of the abdominal subcutis: a case report and literature review. *Int J Hematol.* 2009;89:348-351.
296. Yoshikawa S, Kobayashi H, Kanda S, et al. An Asian variant of intravascular lymphoma diagnosed through splenectomy. *Intern Med*. 2002;41:1215-1216.
297. Yousem SA, Colby TV. Intravascular lymphomatosis presenting in the lung. *Cancer* 1990;65:349-353.
298. Zeidman A, Horowitz A, Fradin Z, Cohen A, Wolfson L, Elimelech O. Fulminant intravascular lymphoma presenting as fever of unknown origin. *Leuk Lymphoma.* 2004;45:1691-1693.
299. Zhao XF, Sands AM, Ostrow PT, Halbiger R, Conway JT, Bagg A. Recurrence of nodal diffuse large B-cell lymphoma as intravascular large B-cell lymphoma: is an intravascular component at initial diagnosis predictive? *Arch Pathol Lab Med.* 2005;129:391-394.
